# Effects of exercise interventions on cognitive function in patients with cognitive dysfunction: an umbrella review of meta-analyses

**DOI:** 10.3389/fnagi.2025.1553868

**Published:** 2025-05-16

**Authors:** Guangyao Sun, Xingyi Ding, Zhong Zheng, Hongtao Ma

**Affiliations:** ^1^College of Education, Beijing Sport University, Beijing, China; ^2^Department of Physical Education, Peking University, Beijing, China

**Keywords:** exercise, cognitive dysfunction, umbrella review, meta-analysis, systematic review

## Abstract

**Objective:**

This umbrella review assessed the quality, potential biases, and effects of exercise interventions on cognitive function in individuals with cognitive impairments.

**Methods:**

A comprehensive umbrella review of meta-analyses of randomized controlled trials (RCTs) was performed to evaluate the effects of exercise on cognitive function in individuals with cognitive impairments. Databases including Web of Science, PubMed, Embase, and the Cochrane Database of Systematic Reviews were searched. Outcomes were evaluated using the Grading of Recommendations, Assessment, Development and Evaluation (GRADE) system, classified as “high,” “moderate,” “low,” or “very low” quality.

**Results:**

A total of 55 meta-analyses were included, covering dementia, cognitive impairment, MCI, Alzheimer’s disease (AD), Parkinson’s disease (PD), and stroke. Cognitive outcomes were assessed using scales like MMSE and MoCA. High-quality evidence supports Exergaming (SMD 0.69), Tai Chi (SMD 0.36), and traditional Chinese mind–body exercises (SMD 0.32) for improving MMSE and MoCA Score in MCI patients. For dementia, moderate-quality evidence shows resistance training (SMD 0.60) and Tai Chi (SMD 0.27) have positive effects. Aerobic exercise (MD 2.95) was more effective for AD, while mind–body exercises (MD 1.68) benefitted PD patients. Multi-component exercises (SMD 0.67) improved MMSE and MoCA scores in post-stroke cognitive impairment. For unspecified cognitive impairments, combining exercise with cognitive training and traditional Chinese exercises showed higher effectiveness. Due to small sample sizes, all findings were Class IV evidence, requiring further research.

**Conclusion:**

Moderate to high-quality evidence supports Exergaming, Tai Chi, and traditional Chinese exercises in improving cognitive function in MCI. For dementia, resistance training and Tai Chi are effective; for AD, aerobic exercise; for PD, mind–body exercises; and for post-stroke cognitive impairment, multi-component exercises are beneficial.

**Systematic review registration:**

https://www.crd.york.ac.uk/PROSPERO/view/CRD42024587635, identifier [CRD42024587635].

## 1 Introduction

Cognitive dysfunction encompasses several cognitive impairments, from mild cognitive impairment (MCI) to dementia, and is also referred to as cognitive decline, deficit, or disability. Diseases causing cognitive dysfunction are categorized into degenerative and non-degenerative types based on their pathogenesis. Degenerative conditions comprise Alzheimer’s disease (AD) and Parkinson’s disease (PD), while non-degenerative conditions include vascular dementia and brain trauma. These conditions pose significant health risks to middle-aged and elderly populations, affecting quality of life and placing a dual burden on social and economic systems (Wang et al., 2019). Currently, approximately 43.8 million people worldwide are affected by cognitive impairments, with 10.427 million in China. It is estimated that by 2050, the global number of affected individuals will reach 152 million (Ren R. J. et al., 2021). Among the Chinese population aged 60 and above, the prevalence of MCI and dementia is 15.5 and 6.0%, respectively (Jia et al., 2020). Cognitive impairment not only contributes to specific diseases such as Alzheimer’s but also interacts with various health conditions and lifestyle factors, becoming a significant indicator of overall health. It is widely believed that cognitive impairments, if left untreated, will progress to dementia, an irreversible neurodegenerative disease with no current treatment options. As cognitive function declines, the burden on individuals, families, and society increases.

In recent years, exercise has emerged as an effective non-pharmacological intervention for improving cognitive impairment, thanks to advancements in basic research. Scholars have emphasized its significant role in preventing and improving cognitive dysfunction (Huang et al., 2020). Numerous randomized controlled trials (RCTs) have demonstrated the positive effects of physical activity on cognitive dysfunction (Mollinedo Cardalda et al., 2019; Langoni et al., 2019) and its ability to enhance brain plasticity (Bolandzadeh et al., 2015; Gajewski and Falkenstein, 2016). However, some studies report that exercise does not significantly improve cognitive impairment (Tang et al., 2016). While several meta-analyses have demonstrated the positive impact of physical activity on cognitive functions in patients with cognitive impairments, factors such as the diverse causes of cognitive dysfunction in study populations, the types and intensities of exercise interventions, and the varying durations of the studies prevent a definitive conclusion. Questions remain regarding the effectiveness of exercise for patients with different types of cognitive impairments, which specific exercises are most effective, and whether the improvements vary across different diseases.

Cognitive impairment is a multifactorial condition, and evaluating intervention measures can provide clinicians with effective prevention strategies. This review examines published systematic reviews and performs a meta-analysis on the effects of physical activity on cognitive function in patients with diverse cognitive impairments. It also employs the AMSTAR 2 tool to appraise the methodological quality of these studies and utilizes the GRADE system to determine the reliability of evidence for the intervention effect estimates.

## 2 Methods and analysis

### 2.1 Design and registration

We conducted a systematic search, extraction, and analysis of data from published meta-analyses on the effects of physical exercise approaches on cognitive performance in patients with cognitive dysfunction, following PRISMA guidelines (Shamseer et al., 2015). This umbrella review followed the methodology specified in the Joanna Briggs Institute Manual for Evidence Synthesis (Aromataris et al., 2022) and the Cochrane Handbook for Systematic Reviews (Cumpston et al., 2019). We also registered it with the International Prospective Register of Systematic Reviews (PROSPERO), under registration number CRD 42024587635.^1^

### 2.2 Eligibility criteria

Meta-analyses of RCTs investigating the impact of exercise interventions on cognitive function in patients with cognitive impairment, regardless of ethnicity or sex, from any country or setting, were eligible for inclusion. When two or more outcome measures were presented in a single meta-analysis, data on the MMSE were extracted. When multiple meta-analyses on the same exercise intervention were published more than 24 months apart, the most recent meta-analysis was included for data analysis. If meta-analyses were published within 24 months of each other, priority was given to the one with the most RCTs. If the number of RCTs was the same, the meta-analysis with the higher AMSTAR score was prioritized. When the most recent meta-analysis lacked a dose–response analysis but another one included it, both were considered for data extraction. Studies published in languages other than English, as well as those involving animal models or cell cultures, were excluded from consideration.

### 2.3 Population

This review examines meta-analyses on the effects of physical activity interventions on cognitive performance in individuals with cognitive impairment. The primary aim of the selected studies was to identify interventions that improve or worsen cognitive function outcomes. Studies evaluating the effect of specific exercises on the risk of cognitive impairment were excluded from the analysis.

### 2.4 Exposure

We incorporated meta-analyses reporting a minimum of one exercise intervention for cognitive impairment, such as aerobic, resistance, or multi-component exercise. The effects on clinical outcomes were evaluated using mean difference (MD), or standard mean difference (SMD) with 95% confidence intervals (CIs).

### 2.5 Results

The outcome scales used in this comprehensive assessment include the Mini-Mental State Examination (MMSE), Montreal Cognitive Assessment (MoCA), Cambridge Cognitive Examination Scale (CAMCOG), AD Assessment Scale-Cognitive Subscale (ADAS-Cog), Rapid Assessment of Cognitive Function (ERFC), Mini-Mental State Examination Cantonese Version (CMMSE), Cornell Depression Rating Scale for Dementia (CSDD), Montreal Cognitive Assessment Korean Version (K-MoCA), Mini-Mental State Examination-Korean Version (MMSE-K), Neurobehavioral Cognitive State Examination (NCSE), Standard Mini-Mental State Examination (SMMSE), Matisse Dementia Rating Scale (MDRS), Parkinson’s Disease Outcome Scale-Cognitive (SCOPA-COG), Parkinson’s Disease Questionnaire-39 (PDQ-39), and Addenbrooke’s Cognitive Examination Revised (ACER).

### 2.6 Study design

Only meta-analyses of RCTs evaluating the impact of physical activity programs on cognitive function in individuals with cognitive impairment, conducted in various countries and regions regardless of race and gender, were included. All included reviews focused on exercise interventions for patients with cognitive impairments and provided detailed descriptions of meta-analysis methods, including search strategies, inclusion/exclusion criteria, quality assessment, outcome evaluation, analysis procedures, and interpretation criteria.

### 2.7 Information sources

A systematic search was conducted in PubMed, Web of Science, the Cochrane Database of Systematic Reviews and Embase until July 2024 to identify relevant systematic reviews and meta-analyses of RCTs. Additionally, reference lists of included meta-analyses were reviewed to locate any additional pertinent articles.

### 2.8 Search strategy

The databases were searched with MeSH terms, keywords, and text relevant to exercise interventions and cognitive dysfunction, following the Scottish Intercollegiate Guidelines Network (SIGN) recommendations for literature search methodology (SIGN, 2020). The detailed PubMed search strategy is as follows: (((“Exercise”[Mesh]) OR (((((((((((((((((((((((((Exercises) OR (Exercise, Physical)) OR (Exercises, Physical)) OR (Physical Exercise)) OR (Physical Exercises)) OR (Physical Activity)) OR (Activities, Physical)) OR (Activity, Physical)) OR (Physical Activities)) OR (Exercise, Aerobic)) OR (Aerobic Exercise)) OR (Aerobic Exercises)) OR (Exercises, Aerobic)) OR (Exercise, Isometric)) OR (Exercises, Isometric)) OR (Isometric Exercises)) OR (Isometric Exercise)) OR (Acute Exercise)) OR (Acute Exercises)) OR (Exercise, Acute)) OR (Exercises, Acute)) OR (Exercise Training)) OR (Exercise Trainings)) OR (Training, Exercise)) OR (Trainings, Exercise))) AND ((“Cognitive Dysfunction”[Mesh]) OR ((((((((((((((((((((((((((Cognitive Dysfunctions) OR (Dysfunction, Cognitive)) OR (Dysfunctions, Cognitive)) OR (Cognitive Disorder)) OR (Cognitive Disorders)) OR (Disorder, Cognitive)) OR (Disorders, Cognitive)) OR (Cognitive Impairments)) OR (Cognitive Impairment)) OR (Impairment, Cognitive)) OR (Impairments, Cognitive)) OR (Mild Cognitive Impairment)) OR (Cognitive Impairment, Mild)) OR (Cognitive Impairments, Mild)) OR (Impairment, Mild Cognitive)) OR (Impairments, Mild Cognitive)) OR (Mild Cognitive Impairments)) OR (Cognitive Decline)) OR (Cognitive Declines)) OR (Decline, Cognitive)) OR (Declines, Cognitive)) OR (Mental Deterioration)) OR (Deterioration, Mental)) OR (Deteriorations, Mental)) OR (Mental Deteriorations)) OR (Cognitive function)))) AND ((systematic review) OR (meta-analysis)). Detailed search terms for the remaining databases are provided in Supplementary Table S1.

### 2.9 Study selection

**Figure 1:**
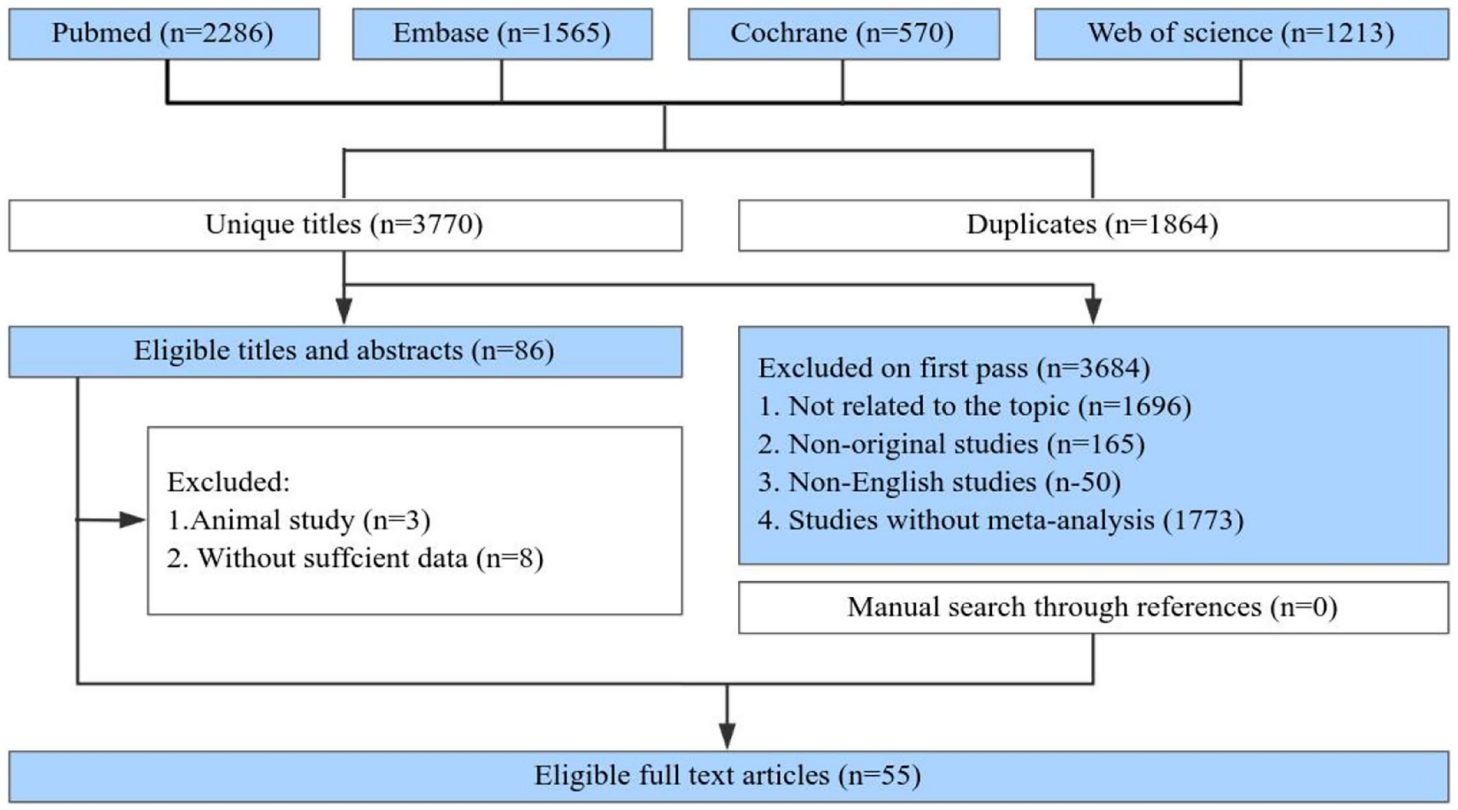
Flowchart of the systematic search and selection process.

Literature screening was performed using Endnote X9. After removing duplicates, two authors independently screened titles, abstracts, and full texts to select meta-analyses that satisfied the inclusion criteria. Any conflicts were resolved through consultation with a third author. Additionally, reference lists were manually searched to detect any meta-analyses potentially missed (Figure 1).

### 2.10 Methodological quality assessment

The methodological quality of each meta-analysis was evaluated by two authors utilizing the AMSTAR tool, a reliable and validated framework for evaluating meta-analyses and systematic reviews (Poole et al., 2017; Shea et al., 2007). The evidence strength for each health outcome was assessed using the GRADE system, with classifications of “high,” “moderate,” “low,” or “very low” (Guyatt et al., 2011). Furthermore, the evidence for each outcome was categorized into five levels: Class I (convincing evidence), Class II (highly suggestive evidence), Class III (suggestive evidence), Class IV (weak evidence), and NS (not significant) (Ioannidis, 2009; Veronese et al., 2018; Wallace et al., 2020; Huang et al., 2023). The classification criteria are provided in Table 1.

**Table 1:** Evidence categories criteria.

Evidence class	Description
Class I: convincing evidence	>1,000 cases (or >20,000 participants for continuous outcomes), statistical significance at *p* < 10^−6^ (random-effects), no evidence of small-study effects and excess significance bias; 95% prediction interval excluded the null, no large heterogeneity (I^2^ < 50%)
Class II: highly suggestive evidence	>1,000 cases (or >20,000 participants for continuous outcomes), statistical significance at *p* < 10^−6^ (random-effects) and largest study with 95% CI excluding the null value
Class III: suggestive evidence	>1,000 cases (or >20,000 participants for continuous outcomes) and statistical significance at *p* < 0.001
Class IV: weak evidence	The remaining significant associations with *p* < 0.05
NS: non-significant	*p* > 0.05

### 2.11 Data extraction

Relevant data from each eligible study were independently extracted by two researchers, and in the event of disagreement, a third author made the decision, including: (1) exercise intervention, (2) control group, (3) disease, (4) outcome, (5) number of studies, (6) author names, (7) sample size, (8) publication date, and (9) MD or SMD estimates with 95% CIs. Additionally, we recorded the meta-analysis model (random or fixed), heterogeneity estimates (I^2^ and Cochran’s Q test), and assessments of small-study bias (Egger and Begg tests, funnel plots). For dose–response and subgroup analyses, we extracted *p* values for nonlinearity and subgroup estimates. Any discrepancies were addressed through consultation with a third author.

### 2.12 Data summary

The MD or SMD with 95% CI was recalculated using either random-effects or fixed-effects models. Heterogeneity was assessed with I^2^ and Cochran’s Q test, while small-study effects were examined with the Egger or Begg tests (for meta-analyses with >10 studies), as appropriate (Theodoratou et al., 2014; Huang Y. et al., 2021; Egger et al., 1997). For dietary interventions rated as Class I-II (high or moderate quality), sensitivity analyses were conducted to evaluate the impact of individual studies on the overall findings. Dose–response analyses were performed for exercise interventions targeting cognitive impairment in patients. In cases where recent meta-analyses excluded clinical studies from earlier analyses, the data were merged and subjected to reanalysis. Heterogeneity was considered significant when *p* < 0.10, and statistical significance for other tests was set at *p* < 0.05. Evidence synthesis was conducted using Review Manager v5.4.1 (Cochrane Collaboration, Oxford, UK), while Egger and Begg tests, as well as sensitivity analyses, were conducted using Stata v15.1.

## 3 Main results

### 3.1 Characteristics of meta-analysis

**Figure 2:**
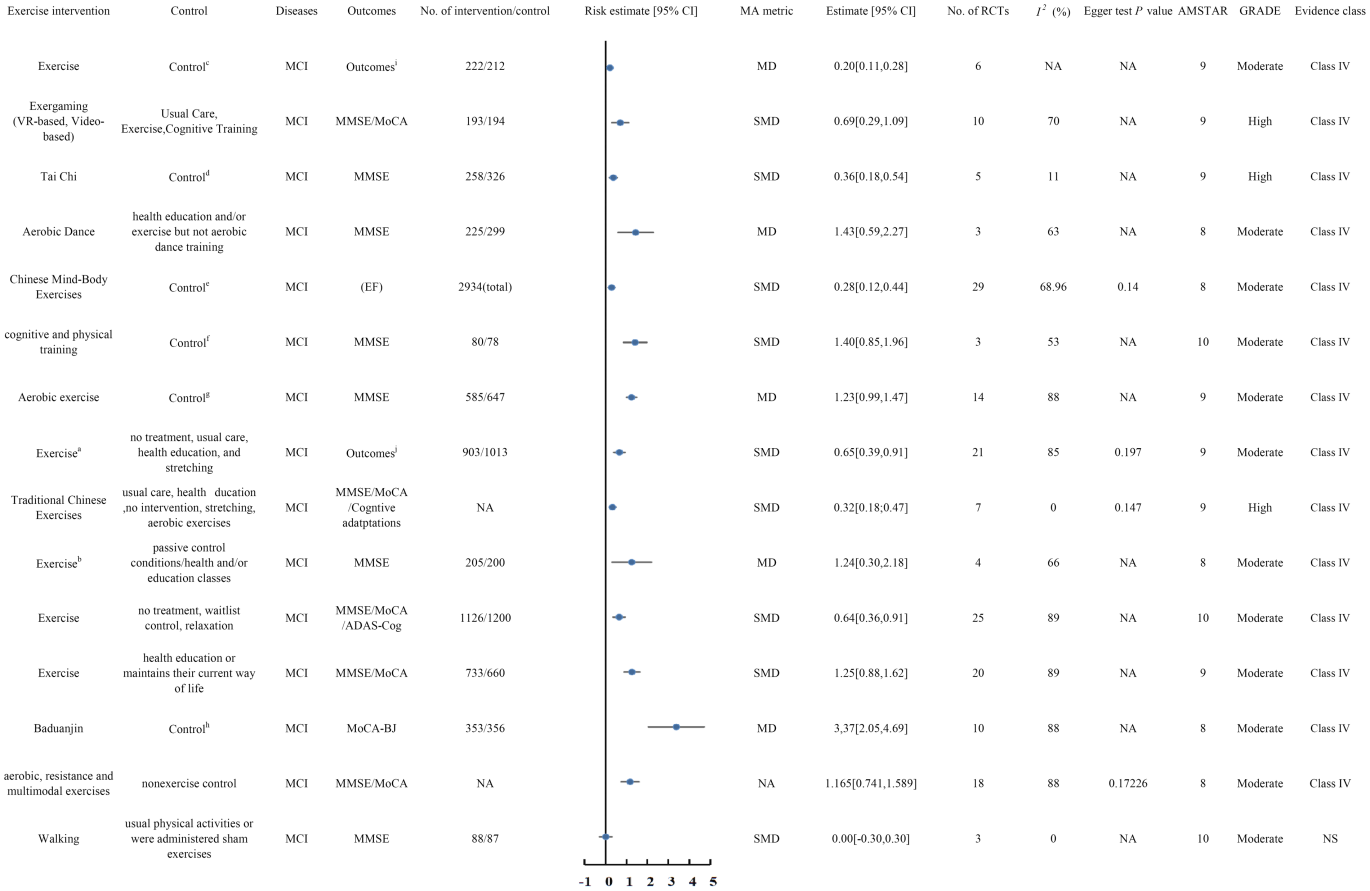
Forest plots of high-quality evidence and moderate-quality evidence showed that exercise intervention can significantly improve clinical outcomes in patients with MCI. CI, confidence interval; AMSTAR, ^a^ measurement tool to assess systematic reviews; GRADE, Grading of Recommendations Assessment, Development, and Evaluation; MMSE, the Mini Mental State Examination; MoCA, the Montreal Cognitive Assessment;CMMSE, Cantonese version of Mini Mental Status Examination; K-MoCA, Korea version of Montreal Cognitive Assessment; MMSE-K, Mini Mental State Examination-Korea version; NCSE, Neurobehavioral Cognitive Status Examination; SMMSE, Standard Mini Mental StateExamination; aaerobic, resistance, multicomponent, and neuromotor exercises ^b^dance/simultaneous multicomponent exercise/momentum-dumbbell training program/exercise training technology ^c^TAU/Daily organized activities/Home safety assessment sessions/Health education classes/Recreational activities/Stretching (HR<50%)/Placebo activity program/Social contacts ^d^the conventional exercise group, patients who received education regarding fall prevention and cognition exercise, and the patient group who were given no treatment ^e^active control group (e.g., physical exercise, educational program, social interaction, cognitive training) or passive control group (e.g., usual care, waitlist control, no intervention) were included. ^f^single cognitive or sham intervention (e.g., placebo control, blank control, and passive control)/two or more control groups (e.g., single physical intervention, single cognitive intervention, or sham intervention) ^g^exercises of stretching, activities of health education, routine care, daily lifestyle, and social recreation ^h^conventional therapy, maintained their daily routine, and did not receive other exercise therapy ^i^the cognitive subscale of the Alzheimer Disease Assessment Scale, the Severe Impairment Battery (for Mini-Mental State Exam [MMSE] score <10), MMSE, or SKT jMMSE/MoCA/CMMSE/K-MoCA/MMSE-K/NCSE/SMMSE.

Figure 1 illustrates the flowchart detailing the literature search and selection process. The systematic search resulted in 5,634 unique articles. Applying our inclusion criteria, 55 meta-analyses were selected. These meta-analyses identified 7 patient categories with cognitive impairment: Mild Cognitive Impairment, Dementia, Alzheimer’s disease, Parkinson’s disease, Stroke, and Mixed Cognitive Impairment. We extracted data on 64 outcomes, of which 57 showed significant associations and 7 did not. After evaluating the evidence quality based on predefined criteria, all results were categorized as Class IV or NS evidence. Based on the GRADE system, 5 exercise interventions were rated as high quality, 28 as moderate, 25 as low, and 5 as very low (Supplementary Table S2). Figure 2 presents the high-and moderate-quality evidence showing that exercise interventions significantly improve clinical outcomes in individuals with cognitive impairment.

### 3.2 MCI

In total, 22 meta-analyses (Chan et al., 2024; Zhou K. et al., 2022; Rampengan et al., 2024; Zhu et al., 2020; Han et al., 2023; Lin et al., 2023; Ren F. F. et al., 2021; Yu et al., 2021; Ströhle et al., 2015; Liu X. et al., 2023; Liu et al., 2024; Han et al., 2022; Ahn and Kim, 2023; Zawaly et al., 2019; Akalp et al., 2024; Wang et al., 2018; Yuan et al., 2022; Shao et al., 2022; Yan et al., 2023; Hewston et al., 2021; Cai et al., 2023; Zhang et al., 2020) have studied the effects of exercise interventions on cognitive function in MCI.

#### 3.2.1 High-quality evidence

There are 3 (Chan et al., 2024; Zhou K. et al., 2022; Rampengan et al., 2024) meta-analyses with high-quality evidence (Figure 2).

##### 3.2.1.1 Exergaming

The meta-analysis conducted by Chan et al. (2024) examined 10 RCTs involving 387 patients with MCI, with Exergaming (both VR-based and Video-based) included as an intervention. The review concluded that Exergaming significantly improved MMSE and MoCA scores (SMD 0.69, 95% CI 0.29 to 1.09) in comparison to Usual Care, Exercise, and Cognitive Training in individuals with MCI. This suggests that Exergaming (VR-based and Video-based) has a beneficial impact on cognitive function in individuals diagnosed with MCI.

##### 3.2.1.2 Traditional Chinese exercises

In 2022, a meta-analysis by Zhou K. et al. (2022) included 7 RCTs, with Traditional Chinese Exercises featured in the pooled analysis. The review found that Traditional Chinese Exercises significantly improved Cognitive Adaptation, MMSE, and MoCA scores (SMD 0.32, 95% CI 0.18 to 0.47) in comparison to usual care, health education, no intervention, stretching, and aerobic exercises in patients with MCI.

##### 3.2.1.3 Tai Chi

The meta-analysis conducted by Rampengan et al. (2024) in 2024 included 5 RCTs with 584 individuals diagnosed with MCI, in which Tai Chi was part of the pooled analysis. The review found that Tai Chi intervention significantly improved MMSE scores, thereby enhancing cognitive function (SMD 0.36, 95% CI 0.18 to 0.54) compared to the conventional exercise group, patients who received fall prevention and cognitive education, and the group that received no treatment, in individuals with MCI.

#### 3.2.2 Moderate-quality evidence

There are 12 meta-analyses (Zhu et al., 2020; Han et al., 2023; Lin et al., 2023; Ren F. F. et al., 2021; Yu et al., 2021; Ströhle et al., 2015; Liu X. et al., 2023; Liu et al., 2024; Han et al., 2022; Ahn and Kim, 2023; Zawaly et al., 2019; Akalp et al., 2024) with moderate-quality evidence (Figure 2).

##### 3.2.2.1 Aerobics

A total of 3 meta-analyses (Zhu et al., 2020; Han et al., 2023; Lin et al., 2023) analyzed the effects of aerobic physical activity interventions on cognitive performance in individuals with MCI. The meta-analysis by Zhu et al. (2020) included 3 RCTs with 524 MCI patients. The comprehensive evaluation showed that Aerobic Dance significantly improved MMSE scores, thereby enhancing cognitive function in MCI patients, compared to standard health education and/or general physical activity, but not compared to aerobic dance training (MD 1.43, 95% CI 0.59 to 2.27).

A meta-analysis by Han et al. (2023) demonstrated that aerobic exercise significantly improved MMSE scores in individuals diagnosed with MCI (MD 1.23, 95% CI 0.99 to 2.47). However, the meta-analysis by Lin et al. (2023) reported that walking had no substantial effect on cognitive function in MCI patients, showing no notable alteration in the MMSE scale (SMD 0, 95% CI −0.30 to 0.30) (Figure 2).

##### 3.2.2.2 Chinese mind–body exercises

Two meta-analyses (Ren F. F. et al., 2021; Yu et al., 2021) have examined the impact of Chinese Mind–Body Exercises on MCI. The meta-analysis by Ren F. F. et al. (2021) included 29 RCTs with 2,934 patients diagnosed with MCI. The review found that Chinese Mind–Body Exercises significantly improved executive function in MCI patients (SMD 0.28, 95% CI 0.12 to 0.44) in comparison to active controls (e.g., cognitive training, educational programs, physical exercise, educational programs, social interaction) or passive controls (e.g., no intervention, usual care, waitlist control).

Yu et al. (2021) found that Baduanjin significantly improved the MoCA-BJ score in MCI patients compared to conventional therapy, maintaining their daily routine without receiving other exercise therapies (MD 3.37, 95% CI 2.05 to 4.69).

##### 3.2.2.3 Exercise OR cognitive and physical training

Four meta-analyses (Ströhle et al., 2015; Liu X. et al., 2023; Liu et al., 2024; Han et al., 2022) have investigated the impact of physical activity on MCI, and one meta-analysis has investigated the impact of combined cognitive and physical training on MCI.

###### 3.2.2.3.1 Exercise

The meta-analysis by Ströhle et al. (2015) analyzed 6 RCTs encompassing 434 individuals diagnosed with MCI. The comprehensive review found that exercise significantly improved cognitive outcomes, including the Alzheimer Disease Assessment Scale cognitive subscale, the Severe Impairment Battery (for MMSE scores <10), MMSE, and SKT scores in MCI patients, compared to treatment as usual (TAU), daily organized activities, home safety assessments, health education classes, recreational activities, stretching (HR < 50%), placebo activity programs, and social interactions (MD 0.20, 95% CI 0.11 to 0.28).

The meta-analysis conducted by Liu X. et al. (2023) included 25 RCTs with 2,326 patients diagnosed with MCI. The review found that exercise significantly improved MMSE, MoCA, and ADAS-Cog scores in MCI patients compared to no treatment, waitlist control, and relaxation (MD 0.64, 95% CI 0.36 to 0.91).

Another meta-analysis conducted by Liu et al. (2024) included 20 RCTs with 1,393 MCI patients. This review demonstrated that exercise significantly improved MMSE and MoCA scores in MCI patients compared to health education or maintaining their current lifestyle (SMD 1.25, 95% CI 0.88 to 1.62). Therefore, exercise can improve cognitive function in individuals with MCI.

###### 3.2.2.3.2 Cognitive and physical training

Han et al. (2022) performed a meta-analysis of 3 RCTs involving 158 MCI patients. The analysis demonstrated that combined cognitive and physical training significantly improved MMSE scores in MCI patients (SMD 1.40, 95% CI 0.85 to 1.96) compared to single cognitive or sham interventions (e.g., placebo, blank, or passive controls) or multiple control groups (e.g., single cognitive or physical interventions, or sham treatments).

##### 3.2.2.4 Multiple exercise

Three meta-analyses (Ahn and Kim, 2023; Zawaly et al., 2019; Akalp et al., 2024) have examined the impact of multiple exercises on cognitive function in patients with MCI. The meta-analysis conducted by Ahn and Kim (2023) included 21 RCTs involving 1,916 MCI patients. The comprehensive evaluation found that aerobic exercise, resistance exercise, multiple exercise types, and neurological exercise significantly improved MMSE, MoCA, CMMSE, K-MoCA, MMSE-K, NCSE, and SMMSE scores in MCI patients compared to usual care, stretching, health education, and no treatment (SMD 0.65, 95% CI 0.39 to 0.91).

The meta-analysis by Zawaly et al. (2019) included 4 RCTs involving 405 patients with MCI. The comprehensive review found that dance, synchronized multi-component exercise, momentum-dumbbell training programs, and sports training techniques had a more notable influence on MMSE scores in individuals diagnosed with MCI compared to passive control conditions, health and/or education courses (MD 1.24, 95% CI 0.30 to 2.18).

Another meta-analysis by Akalp et al. (2024) included 18 RCTs. The comprehensive review showed that aerobic exercise, resistance exercise, and multi-component exercise had more significant effects on MMSE and MoCA scores in MCI patients (SMD 1.165, 95% CI 0.741 to 1.589).

#### 3.2.3 Low-quality evidence

Seven meta-analyses (Wang et al., 2018; Yuan et al., 2022; Shao et al., 2022; Yan et al., 2023; Hewston et al., 2021; Cai et al., 2023; Zhang et al., 2020) provided low-quality evidence, including studies by Wang et al. (2018), Yuan et al. (2022), Shao et al. (2022), Yan et al. (2023), Hewston et al. (2021), Cai et al. (2023), and Zhang et al. (2020). The comprehensive evaluation revealed that Mind–Body Exercise (SMD 0.46, 95% CI 0.6 to 0.85), aerobic dance/square dance/ballroom dance/choreographed exercise (SMD 0.65, 95% CI 0.20 to 1.09), muscle-strengthening activity/aerobic activity/mind–body activity (SMD 0.536, 95% CI 0.371 to 0.70), Multicomponent Exercise (SMD 0.978, 95% CI 0.298 to 1.659), dance (MD 1.58, 95% CI 0.21 to 2.59), mental and physical exercises, including qigong, Ba Duan Jin, taijiquan, yoga, dance, music, and meditation (MD 1.73, 95% CI 0.60 to 2.86), and Resistance Training (SMD 0.53, 95% CI 0.02 to 1.04) all had important effects on the cognitive performance of individuals diagnosed with MCI.

### 3.3 Unclassified cognitive impairment

A total of 4 meta-analyses (Yao et al., 2023; Gu et al., 2021; Wang et al., 2024; Wang et al., 2018) have investigated the impact of exercise on cognitive performance in individuals with cognitive impairment.

#### 3.3.1 High and moderate-quality evidence

**Figure 3:**
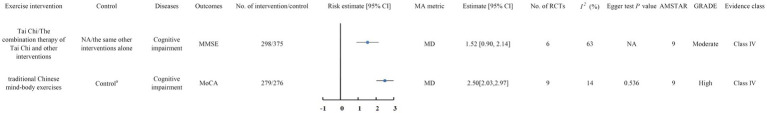
Forest plots of high-quality evidence and moderate-quality evidence showed that exercise interventions can significantly improve clinical outcomes in patients with cognitive impairment. CI, confidence interval; AMSTAR, a measurement tool to assess systematic reviews; GRADE, Grading of Recommendations Assessment, Development, and Evaluation; MMSE, the Mini Mental State Examination; MoCA, the Montreal Cognitive Assessment; ^a^conventional therapy, maintained their daily routine, and did not receive any other exercise therapy.

There are 2 meta-analyses (Yao et al., 2023; Gu et al., 2021) that are high or moderate-quality evidence (Figure 3).

##### 3.3.1.1 Traditional Chinese mind–body exercises

The meta-analysis by Yao et al. (2023) included 9 RCTs with 555 patients diagnosed with cognitive impairment. The comprehensive review found that traditional Chinese mind–body exercises, compared to standard therapy, maintaining usual daily activities and without receiving additional physical activity therapy, significantly improved MMSE scores in patients with cognitive impairment (MD 2.50, 95% CI 2.03 to 2.97) (high-quality evidence).

##### 3.3.1.2 Tai Chi or the combination therapy of Tai Chi and other interventions

The meta-analysis by Gu et al. (2021) included 6 RCTs with 673 patients diagnosed with cognitive impairment. The review found that Tai Chi or the combination of Tai Chi and other programs significantly improved MoCA scores (MD 1.52, 95% CI 0.90 to 2.14) (moderate-quality evidence).

#### 3.3.2 Low-quality evidence

Two meta-analyses (Wang et al., 2024; Wang et al., 2018) with low-quality evidence, namely Wang et al. (2024) and Wang et al. (2018), were included in the evaluation. The comprehensive review found that movement training based on rhythmic auditory stimulation notably enhanced cognitive performance in individuals with cognitive impairment (MD 1.19, 95% CI 0.09 to 2.29). In contrast, Mind–Body Exercise (SMD 0.79, 95% CI −0.09 to 1.67) showed no important effect on cognitive function in these patients.

### 3.4 Dementia

A total of 7 meta-analyses (Yan et al., 2023; de Almeida et al., 2020; Coelho-Junior et al., 2022; Liu D. M. et al., 2023; Li et al., 2019; Chan et al., 2024; Cardona et al., 2021) have investigated the effects of physical activity on cognitive performance in individuals with dementia.

#### 3.4.1 Moderate-quality evidence

**Figure 4:**
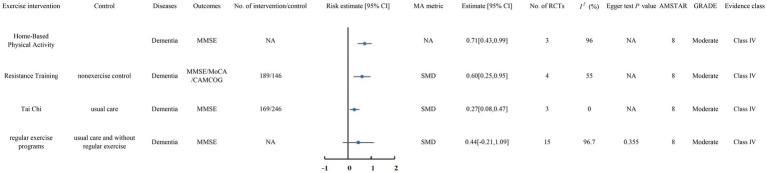
Forest plots of high-quality evidence and moderate-quality evidence showed that exercise interventions can significantly improve clinical outcomes in patients with Dementia. CI, confidence interval; AMSTAR, a measurement tool to assess systematic reviews; GRADE, Grading of Recommendations Assessment, Development, and Evaluation; MMSE, the Mini Mental State Examination; MoCA, the Montreal Cognitive Assessment; CAMCOG, the Cambridge Cognitive Examination.

A total of 4 meta-analyses (de Almeida et al., 2020; Coelho-Junior et al., 2022; Liu D. M. et al., 2023; Li et al., 2019) were classified as moderate-quality evidence (Figure 4).

##### 3.4.1.1 Home-based physical activity

The meta-analysis by de Almeida et al. (2020) included 3 RCTs. The comprehensive review found that Home-Based Physical Activity significantly improved MMSE scores in individuals with dementia (SMD 0.71, 95% CI 0.43 to 0.99).

##### 3.4.1.2 Resistance training

The meta-analysis by Coelho-Junior et al. (2022) included 4 RCTs with 335 patients diagnosed with dementia. The comprehensive review found that resistance training significantly improved MMSE, MoCA, and CAMCOG scores in comparison with non-exercise controls (SMD 0.60, 95% CI 0.25 to 0.95).

##### 3.4.1.3 Tai Chi

The meta-analysis by Liu D. M. et al. (2023) included 3 RCTs with 415 patients diagnosed with dementia. The comprehensive review found that Tai Chi significantly improved MMSE scores in patients compared to usual care (SMD 0.27, 95% CI 0.08 to 0.47).

However, a meta-analysis of 15 RCTs published by Li et al. (2019) showed that regular exercise programs did not significantly improve MMSE scores in patients with dementia compared to usual care and no regular physical activity (SMD 0.44, 95% CI −0.21 to 1.09).

#### 3.4.2 Low and very low-quality evidence

Two meta-analyses (Yan et al., 2023; Chan et al., 2024) provided low-quality evidence, and one meta-analysis (Cardona et al., 2021) provided very low-quality evidence. These studies include Yan et al. (2023), Chan et al. (2024), and Cardona et al. (2021). The comprehensive analysis found that multicomponent exercise (SMD 0.403, 95% CI 0.168 to 0.638) (very low-quality evidence), Exergaming (VR-based, video-based) (SMD 0.38, 95% CI 0.10 to 0.67) (low-quality evidence), and exercise or combined physical and cognitive exercises (SMD 0.48, 95% CI 0.19 to 0.77) (very low-quality evidence) had a significant impact on cognitive performance in patients with dementia.

### 3.5 Alzheimer’s disease

In total, 7 meta-analyses (Zhou S. et al., 2022; Ströhle et al., 2015; Liang et al., 2022; Jia et al., 2019; Roy et al., 2023; Abdullahi et al., 2024; Panza et al., 2018) investigated the impact of physical activity on cognitive performance in individuals with Alzheimer’s disease.

#### 3.5.1 Moderate-quality evidence

**Figure 5:**
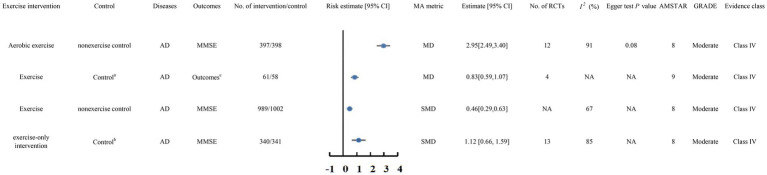
Forest plots of high-quality evidence and moderate-quality evidence showed that exercise interventions can significantly improve clinical outcomes in patients with AD. CI, confidence interval; AMSTAR, a measurement tool to assess systematic reviews; GRADE, Grading of Recommendations Assessment, Development, and Evaluation;AD, Alzheimer’s disease MMSE, the Mini Mental State Examination; MoCA, the Montreal Cognitive Assessment; ^a^TAU/Daily organized activities/Home safety assessment sessions/Health education classes/Recreational activities/Stretching (HR<50%)/Placebo activity program/Social contacts ^b^non-diet, non-exercise control group under the guarantee of basically medical care ^c^the cognitive subscale of the Alzheimer Disease Assessment Scale, the Severe Impairment Battery (for Mini-Mental State Exam [MMSE] score <10), MMSE, or SKT.

A total of 4 meta-analyses (Zhou S. et al., 2022; Ströhle et al., 2015; Liang et al., 2022; Jia et al., 2019) were classified as moderate-quality evidence (Figure 5).

##### 3.5.1.1 Aerobic exercise

The meta-analysis by Zhou S. et al. (2022) included 12 RCTs involving 795 AD patients. The review found that aerobic exercise significantly improved MMSE scores in individuals diagnosed with AD (MD 2.95, 95% CI 2.49 to 3.40).

##### 3.5.1.2 Exercise

Ströhle et al. (2015) included 4 RCTs in their meta-analysis of 119 AD patients. The review found that exercise significantly improved the cognitive subscale of the Alzheimer Disease Assessment Scale, the Severe Impairment Battery (for MMSE scores <10), MMSE, and SKT scores in AD patients compared to TAU, daily organized activities, home safety assessment sessions, health education classes, recreational activities, stretching (HR < 50%), placebo activity programs, and social contacts (MD 0.83, 95% CI 0.59 to 1.07).

A meta-analysis by Liang et al. (2022) involving 1,991 AD patients showed that exercise notably improved MMSE scores in comparison with no exercise (SMD 0.46, 95% CI 0.29 to 0.63).

Additionally, a meta-analysis by Jia et al. (2019) included 13 RCTs involving 681 AD patients. The comprehensive review found that exercise-only interventions significantly improved MMSE scores in AD patients compared to non-diet, non-exercise control groups receiving basic medical care (SMD 1.12, 95% CI 0.66 to 1.59).

#### 3.5.2 Low and very low-quality evidence

Two meta-analyses reported low-quality evidence, and one reported very low-quality evidence. These studies include Roy et al. (2023), Abdullahi et al. (2024), and Panza et al. (2018). The analysis found that aerobic exercise, at-home exercise, and Tai Chi significantly improved cognitive function in AD patients (SMD 0.45, 95% CI 0.07 to 0.83) (low-quality evidence). Similarly, physical activity or exercise delivered at home or via telerehabilitation also had a positive effect (SMD 0.72, 95% CI 0.19 to 1.25) (low-quality evidence). In contrast, combined aerobic and resistance training showed no significant impact on cognitive function (SMD 0.19, 95% CI −0.06 to 0.43) (very low-quality evidence).

### 3.6 Parkinson’s disease

A total of four meta-analyses have investigated the effects of physical activity on cognitive performance in individuals with PD.

#### 3.6.1 Moderate-quality evidence

**Figure 6:**
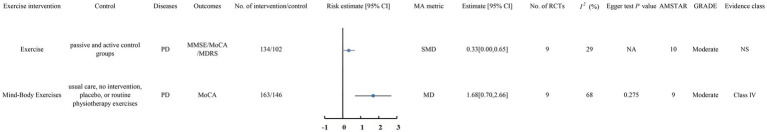
Forest plots of high-quality evidence and moderate-quality evidence showed that exercise interventions can significantly improve clinical outcomes in patients with PD. CI, confidence interval; AMSTAR, a measurement tool to assess systematic reviews; GRADE, Grading of Recommendations Assessment, Development, and Evaluation; PD, Parkinson’s Disease; MMSE, the Mini Mental State Examination; MoCA, the Montreal Cognitive Assessment; MDRS, Mattis Dementia Rating Scale.

There were 2 meta-analyses (Folkerts et al., 2024; Wang et al., 2021) with moderate quality evidence (Figure 6).

##### 3.6.1.1 Exercise

The meta-analysis conducted by Folkerts et al. (2024) included 9 RCTs with 236 PD patients. The comprehensive review found that exercise did not significantly improve MMSE, MoCA, or MDRS scores compared to passive and active control groups (SMD 0.33, 95% CI 0 to 0.65).

##### 3.6.1.2 Mind–body exercises

However, the meta-analysis by Wang et al. (2021) included 9 RCTs with 209 PD patients. The comprehensive review found that mind–body exercises significantly improved MMSE scores in individuals diagnosed PD (MD 1.68, 95% CI 0.70 to 2.66).

#### 3.6.2 Low-quality evidence

Two meta-analyses provided low-quality evidence, namely Kim et al. (2023) and Yin et al. (2023). The comprehensive analysis found that aerobic, strength, balance, flexibility, and combined exercises (SMD 0.69, 95% CI 0.31 to 1.06), as well as Tai Chi (SMD 0.64, 95% CI 0.21 to 1.07), had a significant impact on cognitive function in PD patients.

### 3.7 Stroke

A total of 6 meta-analyses (Zhao et al., 2024; Zhang et al., 2023; Li et al., 2024; Li et al., 2022; Hernandez and Gonzalez-Galvez, 2021; Embrechts et al., 2023) investigated the impact of physical activity on cognitive performance in people with stroke.

#### 3.7.1 Moderate-quality evidence

**Figure 7:**
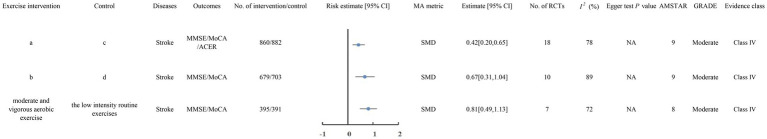
Forest plots of high-quality evidence and moderate-quality evidence showed that exercise interventions can significantly improve clinical outcomes in patients with Stroke. CI, confidence interval; AMSTAR, a measurement tool to assess systematic reviews; GRADE, Grading of Recommendations Assessment, Development, and Evaluation; MMSE, the Mini Mental State Examination; MoCA, the Montreal Cognitive Assessment; ACER, Addenbrooke’s Cognitive Examination-Revised; aresistance training, flexibility training, aerobic training, and mixed training combined with multiple exercises baerobic exercise, resistance exercise, and multiple combination exercises coutine care, conventional physiotherapy, health education, or no treatment droutine non-pharmacological intervention, including a balanced diet, health education, and routine rehabilitation training.

A total of 3 meta-analyses (Zhao et al., 2024; Zhang et al., 2023; Li et al., 2024) were of moderate quality evidence (Figure 7).

##### 3.7.1.1 Multiple combination exercises

The meta-analysis by Zhao et al. (2024) included 18 RCTs with 1,742 stroke patients. The overall review found that aerobic training, flexibility training, resistance training, and combined mixed training with multiple exercises significantly improved MMSE, MoCA, and ACER scores in stroke patients compared to health education, conventional physiotherapy, routine care, or absence of treatment (SMD 0.42, 95% CI 0.20 to 0.65).

The meta-analysis by Zhang et al. (2023) included 10 RCTs with 1,382 stroke patients. The overall evaluation showed that compared to routine non-pharmacological interventions, such as a health education, routine rehabilitation training, a balanced diet, aerobic exercise, resistance exercise, and combined exercises significantly improved MMSE and MoCA scores in stroke patients (SMD 0.67, 95% CI 0.31 to 1.04).

##### 3.7.1.2 Vigorous aerobic exercise

The meta-analysis by Li et al. (2024) included 7 RCTs with 786 stroke patients. The overall evaluation found that moderate and vigorous aerobic exercise significantly improved MMSE and MoCA scores in stroke patients relative to low-intensity routine exercises (SMD 0.81, 95% CI 0.49 to 1.13).

#### 3.7.2 Low-quality evidence

Three meta-analyses provided low-quality evidence, namely Li et al. (2022), Hernandez and Gonzalez-Galvez (2021), and Embrechts et al. (2023). The comprehensive analysis found that aerobic exercise (SMD 0.51, 95% CI 0.16 to 0.86) and exercise (SMD 2.26, 95% CI 1.00 to 3.50) significantly improved cognitive function in stroke patients. Compared to no therapy (SMD 0.43, 95% CI 0.10 to 0.75) and cognitive therapy (SMD 0.18, 95% CI 0.01 to 0.36), both cognitive and motor therapy substantially enhanced cognitive performance in individuals recovering from stroke However, cognitive and motor therapy did not significantly improve cognitive function as opposed to motor therapy alone (SMD 0.18, 95% CI 0 to 0.36).

### 3.8 The mixture of different cognitive impairments

Six meta-analyses investigated the impact of exercise on cognitive performance in individuals with varying cognitive impairments, as well as in those with cognitive impairment alongside healthy individuals.

#### 3.8.1 High and moderate-quality evidence

**Figure 8:**
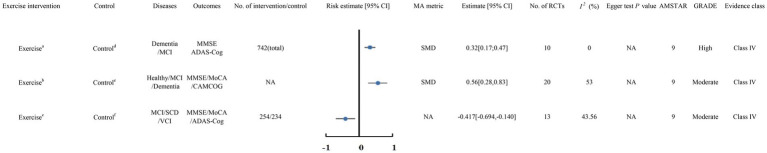
Forest plots of high-quality evidence and moderate-quality evidence showed that exercise interventions can significantly improve clinical outcomes in patients with Stroke. CI, confidence interval; AMSTAR, a measurement tool to assess systematic reviews; GRADE, Grading of Recommendations Assessment, Development, and Evaluation; MCI, mild cognitive impairment; SCD, Subjective Cognitive Decline; VCI, Vscular Cognitive Impairment; MMSE, the Mini Mental State Examination; MoCA, the Montreal Cognitive Assessment; CAMCOG, the Cambridge Cognitive Examination; ADAS-Cog, The Alzheimer’s Disease Assessment Scale–Cognitive Subscale; ^a^combined cognitive and physical exercise training ^b^aerobic exercise and transcranial direct current stimulation ^c^aerobic exercise/resistance training/mind-body exercise ^d^Attentioncontrol educational programmes/Sham cognitive and sham exercise/Treatment as usual/Care as usual/Mock-therapy/Psychosocial support/Education control ^e^aerobic exercise and transcranial direct current stimulation fusual care/lifestyle/sham exercise/health education.

One meta-analysis provided high-quality evidence, and two provided moderate-quality evidence. These meta-analyses evaluated the impact of physical activity on individuals with different cognitive impairments or on both patients with cognitive impairment and healthy individuals (Figure 8).

##### 3.8.1.1 Combined cognitive and physical exercise training

Karssemeijer et al. (2017) conducted a meta-analysis of 10 RCTs involving 742 patients with dementia or MCI. The analysis found that combined cognitive and physical exercise training significantly improved MMSE and ADAS-Cog scores compared to attention control educational programs, sham cognitive or exercise interventions, treatment as usual, care as usual, mock therapy, psychosocial support, or education control (SMD 0.32, 95% CI 0.17 to 0.47) (high-quality evidence).

##### 3.8.1.2 Exercise and transcranial direct current stimulation

The meta-analysis conducted by Talar et al. (2022) included 20 RCTs. The comprehensive review found that exercise combined with transcranial direct current stimulation significantly improved MMSE, MoCA, and CAMCOG scores in healthy individuals, MCI patients, and dementia patients compared to aerobic exercise and transcranial direct current stimulation alone (SMD 0.56, 95% CI 0.28 to 0.83) (moderate-quality evidence).

##### 3.8.1.3 Aerobic exercise or resistance training or mind–body exercise

In addition, the meta-analysis conducted by Karamacoska et al. (2023) included 13 RCTs involving 488 patients with MCI, SCD, and VCI. The comprehensive review found that aerobic exercise, resistance training, or mind–body exercise significantly improved MMSE, MoCA, and ADAS-Cog scores in patients compared to usual care, lifestyle interventions, sham exercise, or health education (SMD −0.417, 95% CI −0.694 to −0.140) (moderate-quality evidence).

#### 3.8.2 Low-quality evidence

Three meta-analyses provided low-quality evidence, namely Sanders et al. (2019), Law et al. (2020), and Huang X. et al. (2021). The comprehensive evaluation found that aerobic, anaerobic, multicomponent, or psychomotor exercise significantly improved cognitive function in individuals with dementia, MCI, or VCI (SMD 0.47, 95% CI 0.19 to 0.74). Exercise also significantly improved cognitive function in individuals with dementia or MCI (SMD 0.44, 95% CI 0.27 to 0.61).

However, one meta-analysis showed that aerobic, multicomponent, mind–body exercise, or resistance exercise did not significantly improve cognitive function in individuals with dementia or MCI (SMD 0.21, 95% CI −0.66 to 1.08).

### 3.9 Heterogeneity

All findings in our study were reassessed through random-or fixed-effects models. Approximately 81% of the results exhibited substantial heterogeneity (I^2^ > 50% or Cochran’s Q test *p* < 0.1). The observed heterogeneity was likely attributable to factors such as study setting, study quality, geographic region, race, age, sex, follow-up duration, sample size, and adjustments for confounding variables.

### 3.10 Risk of bias assessment

Egger’s test was applied in our reanalysis to evaluate publication bias for 58.7% of the results, with 7 showing evidence of bias. Among the results not reanalyzed, 7.7% exhibited publication bias based on statistical tests or funnel plots. The remaining results displayed either an absence of no significant bias or lacked a bias assessment.

### 3.11 AMSTAR score, GRADE, and evidence classification

The median AMSTAR score for all findings was 9 (range 8–10), as detailed in Supplementary Table S3. GRADE ratings indicated that one outcome was downgraded to “moderate” due to imprecision, while others were downgraded to “low” or “very low” because of bias, inconsistency, indirectness, or imprecision. Detailed GRADE classifications are available in Supplementary Table S4. Due to small sample sizes, all outcomes were categorized as IV or NS.

## 4 Discussion

This study included 55 meta-analyses, covering populations with dementia, cognitive impairment, MCI, AD, PD, stroke, and other conditions. Cognitive function was assessed using scales such as MMSE and MoCA. Based on the Cochrane bias risk assessment tool, only 5 of the 55 analyses were of high-quality evidence, while the rest were of moderate quality. The findings indicated that most forms of physical activity improved cognitive performance in individuals with various types of cognitive impairments. However, the effects and mechanisms of different exercise types and modalities on cognitive improvement varied. For patients with MCI, Exergaming, aerobic exercise (moderate-quality evidence), mind–body exercise (moderate-quality evidence), multi-component exercise (moderate-quality evidence), and resistance training (moderate-quality evidence) were found to be more effective. Among these, Exergaming, Tai Chi, and traditional Chinese mind–body exercises were supported by high-quality evidence. For dementia patients, home-based exercise (moderate-quality evidence), resistance training (moderate-quality evidence), Tai Chi (moderate-quality evidence), and regular exercise (moderate-quality evidence) all proved effective. Traditional Chinese mind–body exercises (high-quality evidence) and Tai Chi (moderate-quality evidence) were more effective for patients with cognitive impairments. Aerobic exercise (moderate-quality evidence) was proved to be more effective for AD patients. Mind–body exercise (moderate-quality evidence) had a better effect on PD patients. Multi-component exercise showed greater improvements in cognitive performance for individuals with post-stroke cognitive impairments. For patients with unspecified cognitive impairments, an integration of physical activity and cognitive training (high-quality evidence), aerobic exercise combined with direct current stimulation (moderate-quality evidence), and multi-component exercise (moderate-quality evidence) showed the best effects.

Resistance exercise involves muscle contractions to resist or maintain a specific level of force. Research by Chodzko-Zajko et al. (2009) and Marzetti et al. (2018) has shown that RE is a key therapeutic intervention for mitigating the effects of aging on the neuromuscular system. Resistance training offers several benefits, including improvements in memory and cognitive function, neuroprotective and anti-inflammatory effects, stimulation of neural plasticity, enhanced production of Brain-Derived Neurotrophic Factor (BDNF), and optimization of neurotransmitter balance. It also reduces amyloid protein load and plaques, improves cardiovascular health, optimizes blood circulation, and lowers cardiovascular risk, which helps slow the neurodegenerative process in elderly individuals diagnosed with AD and other forms of dementia (Landrigan et al., 2020). A cross-sectional study has highlighted the correlation between muscle strength and the incidence of MCI, suggesting that resistance training targeting muscle strength could serve as a preventive measure against MCI (Vancampfort et al., 2019). Additionally, studies indicate that resistance training’s potential to enhance cognitive function in MCI patients may be linked to increased levels of Insulin-like Growth Factor-1 (IGF-1), a factor that promotes neuronal growth, survival, and differentiation, thus improving cognitive abilities (Cotman et al., 2007).

Aerobic exercise refers to physical activity that primarily relies on aerobic metabolism to generate the energy needed during exercise. It has gender-specific effects on cognition, glucose metabolism, the hypothalamic–pituitary–adrenal axis, and nutritional activities in patients with cognitive impairments. Moderate-intensity aerobic exercise can enhance overall cognitive performance in individuals with mild cognitive impairment (Song et al., 2018). Research has shown that aerobic exercise significantly increases the levels of BDNF, which is essential for enhancing cognitive abilities and brain development. Dance, as a form of aerobic exercise, is unique due to its requirement for complex and frequent changes in body posture, spatial positioning, steps, and rhythm. These characteristics demand greater attention, memory, control, and execution from participants. Additionally, the combination of dance and music, rich in emotional and spiritual elements, stimulates imagination and perception, further activating various cognitive functions and extensive neural pathways in the brain. During dance interventions, changes in musical rhythm and body positioning activate spatial perception neurons, contributing to brain function remodeling (Doeller et al., 2010; Hafting et al., 2005). Regular limb movements, by increasing levels of BDNF and other growth factors, promote the enhancement of neural structures and delay neurodegenerative changes (Colcombe et al., 2003), thereby improving cognitive abilities (Foster et al., 2011). Magnetic resonance imaging (MRI) studies have shown that dance helps prevent degeneration in the brain’s callosal region. The integrity of white matter in this area is crucial for processing speed (Burzynska et al., 2017), which is essential for cognitive functions and information processing (Salthouse, 1996). Therefore, long-term engagement in dance activities can positively affect cognitive domains including memory, attention, spatial perception, and executive function. These effects are closely linked to brain structure and functional plasticity (Aguiñaga and Marquez, 2017; Ho et al., 2015).

Mind–body exercise is a movement practice focused on emotion and concentration, designed to enhance both physical and mental well-being, reduce stress, and improve body composition. Due to its generally low to moderate intensity and slow pace, it is particularly suitable for individuals with weak constitutions, especially those experiencing cognitive decline or exercise intolerance (Wang et al., 2018). Traditional Chinese mind–body exercises, such as Tai Chi, Qigong and Baduanjin have demonstrated significant improvements in cognitive functions, with high-quality evidence supporting their efficacy. Tai Chi, a central component of traditional Chinese culture (Nyman et al., 2019), integrates the principles of traditional philosophy, including Yin and Yang, the Five Elements (Zou et al., 2019), and meridian theories from traditional Chinese medicine. This practice emphasizes “integrating form and spirit” and the “unity of man and nature,” enhancing mental well-being through physical exercises that incorporate guiding techniques and controlled breathing. From a modern medical perspective, Tai Chi is recognized as an exercise that involves complex movement sequences requiring coordination and precise posture control. Practicing Tai Chi promotes blood circulation, regulates the autonomic nervous system (Sun et al., 2019), strengthens muscles, and improves balance, thereby supporting cognitive function. Furthermore, the meditation and breathing exercises in Tai Chi contribute to stress reduction, alleviating symptoms of anxiety and depression (Yang et al., 2022). This psychological improvement is crucial for maintaining cognitive health.

Multicomponent exercise is a program that incorporates aerobic and resistance training, balance and coordination exercises, and flexibility activities at its core (Tarazona-Santabalbina et al., 2016). Compared to single-mode exercises, combining various exercise modalities has a broader and more beneficial impact on older adults, potentially exerting a greater influence on cognition than either aerobic or strength training alone (Dent et al., 2019). This integrated approach, combining aerobic with strength training, more effectively reduces cardiovascular risk factors. These exercises, by influencing the deposition of abnormal proteins, increasing levels of neurotrophic factors including BDNF and nerve growth factor, improving cerebral blood flow, and reducing systemic inflammation, can prevent neurodegenerative changes and slow cognitive decline in elderly frail patients (Kirk-Sanchez and McGough, 2014). Recent studies have also shown that strength and coordination training positively impact cognitive health (Chang et al., 2012; Voelcker-Rehage et al., 2011). Multicomponent exercises not only improve the logical and memory abilities of older patients with amnestic mild cognitive impairment but also slow the atrophy of the cerebral cortex, significantly benefiting cognitive functions in the elderly (Suzuki et al., 2013). This exercise mode, which integrates aerobic, strength, and balance training, outperforms single aerobic training in significantly enhancing attention and working memory (Smith et al., 2010). Research confirms that structured, personalized, high-intensity, long-duration multicomponent exercise programs help preserve cognitive abilities in older adults (Kirk-Sanchez and McGough, 2014).

Emerging Exergaming methods can enhance cognitive functions by providing diverse cognitive stimuli, promoting cooperation between various brain regions, and improving neuroplasticity. Gallardo et al. further emphasize that precise regulation of exercise dosage is essential for improving cognitive impairments in the elderly. By selecting appropriate exercise dosages for different types of exercises, cognitive levels in the elderly population can be effectively enhanced. In conclusion, the potential improvement mechanisms of different exercise types for patients with different types of cognitive impairment also have their own characteristics (Table 2).

**Table 2:** Underlying biological mechanisms of different locomotion patterns.

Mode of exercise	Underlying biological mechanisms
Aerobic exercise	Increase levels of BDNF
Resistance exercise	Increase levels of IGF-1
Mind–body exercise	Regulates the autonomic nervous system
Multicomponent exercise	Reduces cardiovascular risk factors
Exergaming	Diverse cognitive stimuli

The core pathological mechanisms of different diseases determine their sensitivity to specific exercise modalities. Furthermore, the selection of exercise types is influenced by patients’ motor capacity and intervention objectives. It must be emphasized that significant individual variability exists – the following analysis represents general theoretical considerations that may help delay progression of various symptomatic cognitive impairments, serve preventive functions, and provide clinical references. For MCI patients in early-stage cognitive decline who require both cognitive reserve enhancement and motor function maintenance, exergaming is particularly recommended. This intervention combines physical movement with cognitive tasks (e.g., rapid decision-making, spatial navigation) and dual-task training to activate frontal and hippocampal regions, potentially stimulating neuroplasticity. The interactive design of exergaming may further enhance patient engagement and compliance. Alzheimer’s disease patients frequently exhibit reduced cerebral metabolism and cerebrovascular risks. Aerobic exercise may indirectly preserve cognitive function through comprehensive cardiovascular benefits, potentially improving synaptic plasticity and neurogenesis via enhanced cerebral blood flow, hippocampal volume expansion, and BDNF secretion, thereby potentially slowing neurodegenerative progression. Late-stage dementia patients often experience physical functional decline with elevated fall risks. Resistance training could support neuronal health through increased muscle mass and IGF-1 levels, potentially reducing systemic inflammation. Importantly, muscle strength and daily function maintenance are crucial for preserving independence. Parkinson’s disease patients with motor coordination deficits from nigrostriatal dopamine neuron loss may benefit from Tai Chi’s slow, controlled movements and breathing regulation. This mind–body practice enhances dopaminergic system function, improves balance/gait stability (via basal ganglia-cerebellar circuits), reduces stress hormones (e.g., cortisol), and alleviates rigidity/tremor. Tai Chi’s low-intensity nature minimizes fall risks while its integrated mind–body approach better meets neuromodulatory needs compared to isolated physical training. Post-stroke cognitive impairment often involves motor limitations and heterogeneous regional brain damage. Multicomponent exercise promotes whole-brain network reorganization through multi-neural pathway activation, with diverse exercise modalities enabling adaptive training for different impaired regions. The comprehensive improvements in cardiovascular health, muscular function, and executive functioning make this approach particularly suitable for multidimensional post-stroke recovery. When choosing the right exercise, in addition to the recommendation of the above exercise program, you also need to choose according to your own situation, for the more intense exercise, at the beginning of the contact, you can first reduce its intensity and frequency, and gradually exercise.

To date, numerous researchers globally have conducted clinical studies and evidence-driven studies on the impact of exercise on the cognitive functions of patients with cognitive impairments. This umbrella review assesses the strengths of existing evidence-based approaches to physical activity interventions on the cognitive functions of individuals with cognitive impairments, drawing from systematic reviews and meta-analyses. It provides a comprehensive understanding of potential effective exercise strategies for preventing and improving cognitive functions in these patients, from multiple perspectives. Additionally, it offers a theoretical foundation for developing more clinically effective exercise interventions and provides guidance for future clinical research.

It is important to note that this paper highlights significant heterogeneity in 81% of the results (I^2^ > 50% or Cochran’s Q test *p* < 0.1). The impact of such heterogeneity on effect sizes and conclusions may primarily manifest in the following aspects: High heterogeneity indicates substantial variability in effect sizes across studies, potentially leading to widened confidence intervals for the pooled effect size (e.g., larger 95% CI ranges in certain results). Broader confidence intervals reflect increased uncertainty in the findings. Heterogeneity may arise from differences in exercise intervention types, disease stages, or measurement tools, which could be attributed to variations in intervention formats or baseline cognitive levels among patients, thereby complicating the generalization of conclusions. Additionally, while the study recalculated effect sizes using a random-effects model, high heterogeneity may render the model more sensitive to outliers, undermining the robustness of the results and potentially masking true underlying differences. The translation adheres to academic conventions, preserves statistical terminology (e.g., “random-effects model,” “confidence intervals”), and clarifies causal relationships using phrases like “attributed to” and “thereby.” Complex sentences are structured for readability while maintaining technical precision. Regarding publication bias, the study employed Egger’s test and funnel plots for evaluation, identifying bias in 7 outcomes. Additionally, some unreanalyzed studies may involve underreporting. The implications of publication bias are as follows: If positive results (e.g., studies demonstrating significant cognitive improvement through exercise) are more likely to be published while negative results remain unpublished, meta-analyses may overestimate the true effect, with apparent benefits potentially exaggerated due to the omission of negative studies. Second, if high-quality negative studies are overlooked due to small sample sizes or nonsignificant outcomes, this could lead to underestimation. Furthermore, in contexts of substantial heterogeneity, publication bias may interact with other biases (e.g., selection bias), making it challenging to predict the direction of effect size distortion. This translation maintains precise statistical terminology (e.g., “Egger’s test,” “funnel plots”) and employs academic phrasing (e.g., “omission of negative studies,” “direction of distortion”). Complex causal relationships are clarified through parallel structures (“If…while…”) and logical connectors (“furthermore”), ensuring readability while preserving methodological rigor.

## 5 Limitations

This study has a number of limitations. First, the search was restricted to English-language databases, which may have introduced bias by omitting studies published in other languages. Second, only published data were considered, potentially excluding unpublished or forthcoming evidence. Third, we extracted and analyzed data from existing systematic reviews and meta-analyses, excluding original research not covered in these reviews, as well as studies based on network meta-analyses, due to unclear methods for interpreting network and pairwise meta-analysis results. All included studies were rated as non-significant evidence class. Finally, as this is a triplicate analysis based on meta-analysis, it was not possible to perform subgroup analyses to identify the main components affecting the outcome in question. Despite these limitations, this umbrella review comprehensively documents, for the first time, the existing evidence on the improvement of cognitive functions in patients with cognitive impairments through exercise interventions as reported in previous meta-analyses. By evaluating the strengths and weaknesses of existing evidence-based studies on exercise interventions for improving cognitive functions in patients with cognitive impairments, this article helps clarify exercise strategies for preventing cognitive decline in the elderly. It provides a theoretical basis for designing more effective prevention and management strategies for cognitive impairment in clinical practice and offers guidance for future clinical research. This research followed a rigorous systematic approach. Two independent researchers performed literature searches, screened studies, and extracted relevant data. When adequate information was available, we re-analyzed the mean differences (MD) or SMD with 95% CI using either a random-effects or fixed-effects model. We comprehensively evaluated the heterogeneity and publication bias of each included meta-analysis. Furthermore, three established approaches—AMSTAR, GRADE, and evidence classification criteria—were utilized to assess the methodological rigor and categorize the evidence for each risk factor, allowing us to gauge our confidence in the estimates.

## 6 Conclusion

Moderate to high-quality evidence indicates that Exergaming, Tai Chi, and Traditional Chinese Exercises significantly improve cognitive performance in individuals with MCI. In dementia patients, both resistance training and Tai Chi have shown beneficial effects. Aerobic exercise is particularly effective for individuals with Alzheimer’s disease, while mind–body exercises provide advantages for Parkinson’s disease patients. Additionally, multi-component exercises are beneficial for those with cognitive impairments following a stroke. These findings highlight the positive effect of tailored exercise programs on cognitive performance across various cognitive disorders. However, this review has limitations, including potential bias in original data, heterogeneity, and the overall lower quality of some studies. Therefore, further prospective studies are necessary to confirm the impact of various types of physical activity on cognitive performance improvement and assess their safety in treating cognitive impairments caused by various etiologies. Such studies will provide the latest and comprehensive evidence for guiding exercise interventions aimed at improving cognitive functions in patients with cognitive impairments in clinical settings.

## Data Availability

The original contributions presented in the study are included in the article/Supplementary material, further inquiries can be directed to the corresponding author.

## Footnotes

^1^ https://www.crd.york.ac.uk/PROSPERO/
