# Supplementary material for: Effects of exercise interventions on cognitive function in patients with cognitive dysfunction: an umbrella review of meta-analyses
Source: Front Aging Neurosci. 2025 May 16;17:1553868. doi: 10.3389/fnagi.2025.1553868 (PMC12122535; doi:10.3389/fnagi.2025.1553868)
Supplement: Supplementary file 1 [file Data_Sheet_1.doc]

# Table S1 Literature Search Strategy

# Exercise

Exercises

Exercise, Physical

Exercises, Physical

Physical Exercise

Physical Exercises

Physical Activity

Activities, Physical

Activity, Physical

Physical Activities

Exercise, Aerobic

Aerobic Exercise

Aerobic Exercises

Exercises, Aerobic

Exercise, Isometric

Exercises, Isometric

Isometric Exercises

Isometric Exercise

Acute Exercise

Acute Exercises

Exercise, Acute

Exercises, Acute

Exercise Training

Exercise Trainings

Training, Exercise

Trainings, Exercise

# Cognitive Dysfunction

Cognitive Dysfunctions

Dysfunction, Cognitive

Dysfunctions, Cognitive

Cognitive Disorder

Cognitive Disorders

Disorder, Cognitive

Disorders, Cognitive

Cognitive Impairments

Cognitive Impairment

Impairment, Cognitive

Impairments, Cognitive

Mild Cognitive Impairment

Cognitive Impairment, Mild

Cognitive Impairments, Mild

Impairment, Mild Cognitive

Impairments, Mild Cognitive

Mild Cognitive Impairments

Cognitive Decline

Cognitive Declines

Decline, Cognitive

Declines, Cognitive

Mental Deterioration

Deterioration, Mental

Deteriorations, Mental

Mental Deteriorations

Cognitive function

PUBMED-2286

Search: ((("Exercise"[Mesh]) OR (((((((((((((((((((((((((Exercises) OR (Exercise, Physical)) OR (Exercises, Physical)) OR (Physical Exercise)) OR (Physical Exercises)) OR (Physical Activity)) OR (Activities, Physical)) OR (Activity, Physical)) OR (Physical Activities)) OR (Exercise, Aerobic)) OR (Aerobic Exercise)) OR (Aerobic Exercises)) OR (Exercises, Aerobic)) OR (Exercise, Isometric)) OR (Exercises, Isometric)) OR (Isometric Exercises)) OR (Isometric Exercise)) OR (Acute Exercise)) OR (Acute Exercises)) OR (Exercise, Acute)) OR (Exercises, Acute)) OR (Exercise Training)) OR (Exercise Trainings)) OR (Training, Exercise)) OR (Trainings, Exercise))) AND (("Cognitive Dysfunction"[Mesh]) OR ((((((((((((((((((((((((((Cognitive Dysfunctions) OR (Dysfunction, Cognitive)) OR (Dysfunctions, Cognitive)) OR (Cognitive Disorder)) OR (Cognitive Disorders)) OR (Disorder, Cognitive)) OR (Disorders, Cognitive)) OR (Cognitive Impairments)) OR (Cognitive Impairment)) OR (Impairment, Cognitive)) OR (Impairments, Cognitive)) OR (Mild Cognitive Impairment)) OR (Cognitive Impairment, Mild)) OR (Cognitive Impairments, Mild)) OR (Impairment, Mild Cognitive)) OR (Impairments, Mild Cognitive)) OR (Mild Cognitive Impairments)) OR (Cognitive Decline)) OR (Cognitive Declines)) OR (Decline, Cognitive)) OR (Declines, Cognitive)) OR (Mental Deterioration)) OR (Deterioration, Mental)) OR (Deteriorations, Mental)) OR (Mental Deteriorations)) OR (Cognitive function)))) AND ((systematic review) OR (meta-analysis))

EMBASE-1565


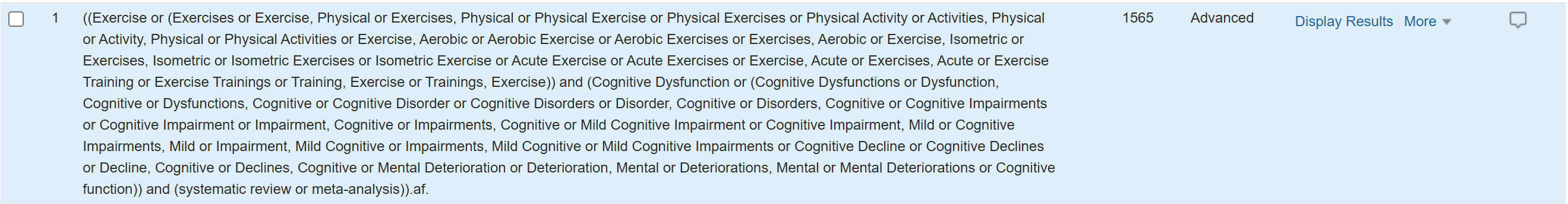


Cochrane Database of Systematic Reviews-570


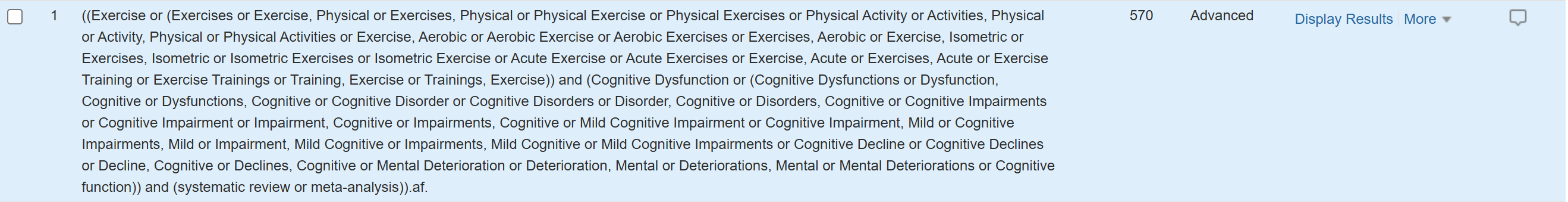


WOS-1213

(((Exercise) OR (((((((((((((((((((((((((Exercises) OR (Exercise, Physical)) OR (Exercises, Physical)) OR (Physical Exercise)) OR (Physical Exercises)) OR (Physical Activity)) OR (Activities, Physical)) OR (Activity, Physical)) OR (Physical Activities)) OR (Exercise, Aerobic)) OR (Aerobic Exercise)) OR (Aerobic Exercises)) OR (Exercises, Aerobic)) OR (Exercise, Isometric)) OR (Exercises, Isometric)) OR (Isometric Exercises)) OR (Isometric Exercise)) OR (Acute Exercise)) OR (Acute Exercises)) OR (Exercise, Acute)) OR (Exercises, Acute)) OR (Exercise Training)) OR (Exercise Trainings)) OR (Training, Exercise)) OR (Trainings, Exercise))) AND ((Cognitive Dysfunction) OR ((((((((((((((((((((((((((Cognitive Dysfunctions) OR (Dysfunction, Cognitive)) OR (Dysfunctions, Cognitive)) OR (Cognitive Disorder)) OR (Cognitive Disorders)) OR (Disorder, Cognitive)) OR (Disorders, Cognitive)) OR (Cognitive Impairments)) OR (Cognitive Impairment)) OR (Impairment, Cognitive)) OR (Impairments, Cognitive)) OR (Mild Cognitive Impairment)) OR (Cognitive Impairment, Mild)) OR (Cognitive Impairments, Mild)) OR (Impairment, Mild Cognitive)) OR (Impairments, Mild Cognitive)) OR (Mild Cognitive Impairments)) OR (Cognitive Decline)) OR (Cognitive Declines)) OR (Decline, Cognitive)) OR (Declines, Cognitive)) OR (Mental Deterioration)) OR (Deterioration, Mental)) OR (Deteriorations, Mental)) OR (Mental Deteriorations)) OR (Cognitive function)))) AND ((systematic review) OR (meta-analysis)) (Abstract)

(((Exercise) OR (((((((((((((((((((((((((Exercises) OR (Exercise, Physical)) OR (Exercises, Physical)) OR (Physical Exercise)) OR (Physical Exercises)) OR (Physical Activity)) OR (Activities, Physical)) OR (Activity, Physical)) OR (Physical Activities)) OR (Exercise, Aerobic)) OR (Aerobic Exercise)) OR (Aerobic Exercises)) OR (Exercises, Aerobic)) OR (Exercise, Isometric)) OR (Exercises, Isometric)) OR (Isometric Exercises)) OR (Isometric Exercise)) OR (Acute Exercise)) OR (Acute Exercises)) OR (Exercise, Acute)) OR (Exercises, Acute)) OR (Exercise Training)) OR (Exercise Trainings)) OR (Training, Exercise)) OR (Trainings, Exercise))) AND ((Cognitive Dysfunction) OR ((((((((((((((((((((((((((Cognitive Dysfunctions) OR (Dysfunction, Cognitive)) OR (Dysfunctions, Cognitive)) OR (Cognitive Disorder)) OR (Cognitive Disorders)) OR (Disorder, Cognitive)) OR (Disorders, Cognitive)) OR (Cognitive Impairments)) OR (Cognitive Impairment)) OR (Impairment, Cognitive)) OR (Impairments, Cognitive)) OR (Mild Cognitive Impairment)) OR (Cognitive Impairment, Mild)) OR (Cognitive Impairments, Mild)) OR (Impairment, Mild Cognitive)) OR (Impairments, Mild Cognitive)) OR (Mild Cognitive Impairments)) OR (Cognitive Decline)) OR (Cognitive Declines)) OR (Decline, Cognitive)) OR (Declines, Cognitive)) OR (Mental Deterioration)) OR (Deterioration, Mental)) OR (Deteriorations, Mental)) OR (Mental Deteriorations)) OR (Cognitive function)))) AND ((systematic review) OR (meta-analysis))
