# Supplementary material for: Effects of exercise interventions on cognitive function in patients with cognitive dysfunction: an umbrella review of meta-analyses
Source: Front Aging Neurosci. 2025 May 16;17:1553868. doi: 10.3389/fnagi.2025.1553868 (PMC12122535; doi:10.3389/fnagi.2025.1553868)
Supplement: Supplementary file 2 [file Data_Sheet_2.doc]

TableS2. Effects of dietary intervention on diabetic nephropathy.

| Exercise intervention | Control | Diseases | Outcomes | Total eligible MA | Included MA | Sample size intervention/control | MA metric | Estimates [95% CI] | No. of RCTs | Effects model | *I*^2^; *Q* test *P* value | Egger test *P* value | AMSTAR | Evidence class | GRADE |
| --- | --- | --- | --- | --- | --- | --- | --- | --- | --- | --- | --- | --- | --- | --- | --- |
| ***Significant associations*** | | | | | | | | | | | | | | | |
| Video Games | a control condition any cognitive stimulation/training technique | AD/MCI | MMSE | 1 | Ferreira-Brito2021 | 95/83 | MD | 1.64[0.60,2.69] | 4 | Random | 0%；0.52 | NA | 9 | NS | Low |
| Aerobic exercise |  | AD | MMSE | 3 | Zhou2022 | 397/398 | MD | 2.95[2.49,3.40] | 12 | Fixed | 91%,<0.00001 | 0.08 | 8 | Class IV | Moderate |
| Exercise | TAU/Daily organized activities/Home safety assessment sessions/Health education classes/Recreational activities/Stretching (HR<50%)/Placebo activity program/Social contacts | AD | the cognitive subscale of the Alzheimer Disease Assessment Scale, the Severe Impairment Battery (for Mini-Mental State Exam [MMSE] score <10), MMSE, or SKT | 1 | Strohle2015 | 61/58 | MD | 0.83[0.59,1.07] | 4 | Fixed | NA,<0.05 | NA | 9 | Class IV | Moderate |
| Exercise | TAU/Daily organized activities/Home safety assessment sessions/Health education classes/Recreational activities/Stretching (HR<50%)/Placebo activity program/Social contacts | MCI | the cognitive subscale of the Alzheimer Disease Assessment Scale, the Severe Impairment Battery (for Mini-Mental State Exam [MMSE] score <10), MMSE, or SKT | 5 | Strohle2015 | 222/212 | MD | 0.20[0.11,0.28] | 6 | Fixed | NA,0.01 | NA | 9 | Class IV | Moderate |
| Exercise | nonexercise control | AD | MMSE | 2 | Liang2022 | 989/1002 | SMD | 0.46[0.29,0.63] | NA | Random | 67%,<0.01 | NA | 8 | Class IV | Moderate |
| TCM exercise therapy | maintaining the same lifestyle, health education, routine care, low-intensity stretching and stretching exercises. | AD/MCI | MMSE/MoCA | 1 | Guo2024 | NA | SMD | 0.83[0.62,1.04] | 20 | Random | 78.4%,0.000 | <0.001 | 9 | Class IV | Very low |
| aerobic exercise, athome exercise, Tai Chi | conventional drugs | AD | MMSE | 1 | Roy2023 | 340/291 | SMD | 0.45[0.07,0.83] | 7 | Random | 78.5%,0.000 | 0.260 | 8 | Class IV | Low |
| physical activity or exercise carried out at home or delivered via telerehabilitation | usual care or another form of exercise | AD | MMSE | 2 | Abdullahi2024 | 41/43 | SMD | 0.72[0.19,1.25] | 3 | Random | 28%,0.25 | NA | 10 | Class IV | Low |
| exercise-only intervention | non-diet, non-exercise control group under the guarantee of basically medical care | AD | MMSE | 1 | Jia2019 | 340/341 | SMD | 1.12 [0.66, 1.59] | 13 | Random | 85%,0.000 | NA | 8 | Class IV | Moderate |
| exercise, aerobic activity, physical exercise | social visit or activity, or usual treatment or care | AD/Dementia |  | 1 | Zeng2023 | 766/600 | SMD | 0.23[0.05,0.41] | 18 | Random | 57.2%,0.001 | <0.05 | 9 | Class IV | Very low |
| Tai Chi and cognitive interventions | Muscle stretching and toning exercise/Attention control/Health education/TAU/Heath advice | Cognitive impairment/MCI | MMSE/MoCA/ADAS-Cog/Clinical Dementia Rating/Dementia Rating Scale | 1 | Li2022 | 297/371 | SMD | 0.74[0.19,1.29] | 6 | Random | 89%,<0.01 | NA | 8 | Class IV | Low |
| Tai Chi/The combination therapy of Tai Chi and other interventions | NA/the same other interventions alone | Cognitive impairment | MMSE | 1 | Gu2021 | 298/375 | MD | 1.52 [0.90, 2.14] | 6 | Random | 63%,0.02 | NA | 9 | Class IV | Moderate |
| movement training based on rhythmic auditory stimulation | conventional treatment, music listening, cognitive training, etc. | Cognitive impairment | MMSE | 1 | Wang2024 | 222/228 | MD | 1.19[0.09,2.29] | 6 | Random | 73%,0.002 | NA | 10 | Class IV | Low |
| traditional Chinese mind-body exercises | conventional therapy, maintained their daily routine, and did not receive any other exercise therapy | Cognitive impairment | MoCA | 1 | Yao2023 | 279/276 | MD | 2.50[2.03,2.97] | 9 | Fixed | 14%,0.32 | 0.536 | 9 | Class IV | High |
| aerobic/anaerobic/multicomponent /psychomotor exercise | nonphysical activity or stretching and toning | Dementia/MCI/VCI | MMSE | 1 | Sanders2019 | 699(total) |  | 0.47[0.19,0.74] | 9 | multilevel mixed-effects models | 1.05,>0.05 | 1.77 | 8 | Class IV | Low |
| Multicomponent Exercise | no treatment/usual care/placebo/other conservative treatments | Dementia | MMSE/ERFC/ADAS-cog | 2 | Yan2023 | 864(total)\ | SMD | 0.403[0.168-0.638] | 9 | Random | 64%,<0,05 | 0.004 | 9 | Class IV | Very low |
| Mind-Body Exercise | nonexercise control | MCI | MMSE | 2 | Wang2018 | 305/379 | SMD | 0.46[0.06,0.85] | 4 | Random | 84%,0.0003 | 0.267 | 8 | Class IV | Low |
| Exergaming (VR-based, Video-based) | Usual Care, Exercise,Cognitive Training | MCI | MMSE/MoCA | 1 | Chan2024 | 193/194 | SMD | 0.69[0.29,1.09] | 10 | Random | 70%,0.0005 | NA | 9 | Class IV | High |
| Exergaming (VR-based, Video-based) | Usual Care, Exercise,Cognitive Training | Dementia | MMSE/MoCA | 1 | Chan2024 | 117/85 | SMD | 0.38[0.10,0.67] | 4 | Random | 0%,0.43 | NA | 9 | Class IV | Low |
| Home-Based Physical Activity |  | Dementia | MMSE | 2 | de Almeida2020 |  |  | 0.71[0.43,0.99] | 3 |  | 96%,0.00 | NA | 8 | Class IV | Moderate |
| Exercise/combining physical and cognitive exercises | usual care, social activities, or handicrafts | Dementia | MMSE/ADAS-Cog | 3 | Cardona2021 | 610/763 | SMD | 0.48[0,19,0.77] | 16 | Random | 86%.≤0.0001 | NA | 8 | Class IV | Very low |
| Exercise | nonexercise control | Dementia/MCI | MMSE | 3 | Law2020 | 2079(total) | SMD | 0.44[0.27,0.61] | 26 | Random | 69% | <0.1 | 9 | Class IV | Low |
| combined cognitive and physical exercise training | Attentioncontrol educational programmes/Sham cognitive and sham exercise/Treatment as usual/Care as usual/Mock-therapy/Psychosocial support/Education control | Dementia/MCI | MMSE/ADAS-Cog | 1 | Karssemeije2017 | 742(total) | SMD | 0.32[0.17;0.47] | 10 | Random | 0%,>0.05 | NA | 9 | Class IV | High |
| Resistance Training | nonexercise control | Dementia | MMSE/MoCA/CAMCOG | 1 | Coelho-Junior2022 | 189/146 | SMD | 0.60[0.25,0.95] | 4 | Random | 55%,0.02 | NA | 8 | Class IV | Moderate |
| Tai Chi | usual care | Dementia | MMSE | 1 | Liu2023 | 169/246 | SMD | 0.27[0.08,0.47] | 3 | Random | 0%.0.58 | NA | 8 | Class IV | Moderate |
| aerobic exercise and transcranial direct current stimulation | aerobic exercise and transcranial direct current stimulation | Healthy, MCI and Dementia | MMSE/MoCA/CAMCOG | 1 | Talar2022 |  | SMD | 0.56[0.28,0.83] | 20 | Random | 53%,0.01 | NA | 9 | Class IV | Moderate |
| aerobic dance/square dance/ballroom dance/choreographed exercise | Physical therapy/Usual practice/Health education/Blank control/Usual care/Usual lifestyle/Regular care/Medicine | MCI | MMSE | 1 | Yuan2022 | 278/280 | SMD | 0.65[0.20,1.09] | 8 | Random | 84%,<0.00001 | 0.755 | 8 | Class IV | Low |
| aerobic exercise/resistance training/mind-body exercise | usual care/lifestyle/sham exercise/health education | MCI/SCD/VCI | MMSE/MoCA/ADAS-Cog | 1 | Karamacoska2023 | 254/234 |  | -0.417[-0.694,-0.140] | 13 | Random | 43.56%,0.003 | NA | 9 | Class IV | Moderate |
| muscle-strengthening activity/aerobic activity/mind- body activity | health education/social activities/active controls | MCI | MMSE | 1 | Shao2022 | 836/852 |  | 0.536[0.371,0.701] | 24 | Random | 59%.0.000 | <0.05 | 9 | Class IV | Low |
| Multicomponent Exercise | no treatment/usual care/placebo/other conservative treatments | MCI | MMSE/MoCA/ADAS-Cog | 3 | Yan2023 | 384(total)\ | SMD | 0.978[0.298,1.659 | 10 | Random | 93%,<0,05 | 0.303 | 9 | Class IV | Low |
| Tai Chi | the conventional exercise group, patients who received education regarding fall prevention and cognition exercise, and the patient group who were given no treatment | MCI | MMSE | 2 | Rampengan2024 | 258/326 | SMD | 0.36[0.18,0.54] | 5 | Random | 11%,0.34 | NA | 9 | Class IV | High |
| Aerobic Dance | health education and/or exercise but not aerobic dance training | MCI | MMSE | 1 | Zhu2020 | 225/299 | MD | 1.43[0.59,2.27] | 3 | Random | 63%,0.07 | NA | 8 | Class IV | Moderate |
| Chinese Mind-Body Exercises | active control group (e.g., physical exercise, educational program, social interaction, cognitive training) or passive control group (e.g., usual care, waitlist control, no intervention) were included. | MCI | (EF) | 1 | Ren2021 | 2934(total) | SMD | 0.28[0.12,0.44] | 29 | Random | 68.96%,<0.001 | 0.14 | 8 | Class IV | Moderate |
| cognitive and physical training | single cognitive or sham intervention (e.g., placebo control, blank control, and passive control)/two or more control groups (e.g., single physical intervention, single cognitive intervention, or sham intervention) | MCI | MMSE | 2 | Han2022 | 80/78 | SMD | 1.40[0.85,1.96] | 3 | Random | 53%,0.15 | NA | 10 | Class IV | Moderate |
| Dance | education, walking, waitlisted or no physical activity | MCI | MMSE | 1 | Hewston2021 | 156/132 | MD | 1.58[0.21,2.95] | 2 | Random | 79%,0.03 | NA | 10 | Class IV | Low |
| Aerobic exercise | exercises of stretching, activities of health education, routine care, daily lifestyle, and social recreation | MCI | MMSE | 3 | Han2023 | 585/647 | MD | 1.23[0.99,1.47] | 14 | Fixed | 88%,<0.00001 | NA | 9 | Class IV | Moderate |
| aerobic, resistance, multicomponent, and neuromotor exercises | no treatment, usual care, health education, and stretching | MCI | MMSE/MoCA/CMMSE/K-MoCA/MMSE-K/NCSE/SMMSE | 1 | Ahn2023 | 903/1013 | SMD | 0.65[0.39,0.91] | 21 | Random | 85%,<0.00001 | 0.197 | 9 | Class IV | Moderate |
| physical and mental exercises such as taijiquan, Ba Duan Jin, qigong, meditation, yoga, music and dance | conventional care, health education or blank | MCI | MMSE | 1 | Cai2023 | 668/800 | MD | 1.73[0.60,2.86] | 14 | Random | 96%,<0.00001 | 0.072 | 9 | Class IV | Low |
| Traditional Chinese Exercises | usual care, health education ,no intervention, stretching, aerobic exercises | MCI | MMSE/MoCA/Cogntive adatptations | 1 | Zhou2022 |  | SMD | 0.32[0.18;0.47] | 7 | Random | 0.0%,0.517 | 0.147 | 9 | Class IV | High |
| dance/simultaneous multicomponent exercise/momentum-dumbbell training program/exercise training technology | passive control conditions/health and/or education classes | MCI | MMSE | 1 | Zawaly2022 | 205/200 | MD | 1.24[0.30,2.18] | 4 | Random | 66%,0.03 | NA | 8 | Class IV | Moderate |
| Resistance Training | routine lifestyle without any exercise activities, balance and tone exercise, and sham training similar to the resistance training | MCI | MMSE/MoCA/ADAS-Cog | 1 | Zhang2020 | 95/87 | SMD | 0.53[0.02,1.04] | 5 | Random | 62%,0.03 | NA | 8 | Class IV | Low |
| Exercise | no treatment, waitlist control, relaxation | MCI | MMSE/MoCA/ADAS-Cog |  | Liu2023 | 1126/1200 | SMD | 0.64[0.36,0.91] | 25 | Random | 89%,<0.00001 | NA | 10 | Class IV | Moderate |
| Exercise | health education or maintains their current way of life | MCI | MMSE/MoCA | 5 | Liu2024 | 733/660 | SMD | 1.25[0.88,1.62] | 20 | Random | 89%,<0.00001 | NA | 9 | Class IV | Moderate |
| Baduanjin | conventional therapy, maintained their daily routine, and did not receive other exercise therapy | MCI | MoCA-BJ | 1 | Yu2021 | 353/356 | MD | 3,37[2.05,4.69] | 10 | Random | 88%,<0.00001 | NA | 8 | Class IV | Moderate |
| aerobic, resistance and multimodal exercises |  | MCI | MMSE/MoCA | 1 | Akalp2024 |  |  | 1.165[0.741,1.589] | 18 | Random | 88%,<0.001 | 0.17226 | 8 | Class IV | Moderate |
| Exercise | passive and active control groups | PD | MMSE/MoCA/MDRS | 1 | Folkerts2024 | 134/102 | SMD | 0.33[0.00,0.65] | 9 | Random | 29%，0.19 | NA | 10 | NS | Moderate |
| aerobic/strength/balance exercise/flexibility exercise/combined exercise | Usual care/No intervention/Stretching/Wait-list | PD | MMSE/MoCA/SCOPA-COG | 1 | Kim2023 |  | SMD | 0.69[0.31,1.06] | 16 | Random | 76.16%,<0.001 | 0.02 | 9 | Class IV | Low |
| Mind-Body Exercises | usual care, no intervention, placebo, or routine physiotherapy exercises | PD | MoCA | 1 | Wang2021 | 163/146 | MD | 1.68[0.70,2.66] | 9 | Random | 68%,0.002 | 0.275 | 9 | Class IV | Moderate |
| Tai Chi | other training forms, usual healthcare, or no intervention | PD | MoCA/K-MMSE/PDQ-39 | 1 | Yin2023 | 322/249 | SMD | 0.64[0.21,1.07] | 6 | Random | 82%,<0.00001 | NA | 8 | Class IV | Low |
| Aerobic exercise |  | Post-stroke | MMSE/MoCA | 1 | Li2022 | 273/281 | SMD | 0.51[0.16,0.86] | 6 | Random | 66%.0.007 | NA | 9 | Class IV | Low |
| resistance training, flexibility training, aerobic training, and mixed training combined with multiple exercises | outine care, conventional physiotherapy, health education, or no treatment | Post-stroke | MMSE/MoCA/ACER | 1 | Zhao2024 | 860/882 | SMD | 0.42[0.20,0.65] | 18 | Random | 78%,<0.00001 | NA | 9 | Class IV | Moderate |
| aerobic exercise, resistance exercise, and multiple combination exercises | routine non-pharmacological intervention, including a balanced diet, health education, and routine rehabilitation training | Post-stroke | MMSE/MoCA | 1 | Zhang2023 | 679/703 | SMD | 0.67[0.31,1.04] | 10 | Random | 89%,<0.00001 | NA | 9 | Class IV | Moderate |
| Exercise | nonexercise control | Stroke | MMSE/MoCA | 1 | Hernandez2021 | 309/323 | SMD | 2.26[1.0,3.5] | 9 | Random/Fixed | 97%,0.00028 | <0.05 | 8 | Class IV | Low |
| CMT | No Therapy | Stroke |  | 1 | Embrechts2023 |  |  | 0.43[0.10,0.75] | 7 | Random | 60.90% | NA | 9 | NS | Low |
| CMT | MT | Stroke |  | 1 | Embrechts2023 |  |  | 0.18[0.00,0.36] | 18 | Random | 63.10% |  | 9 |  | Low |
| CMT | CT | Stroke |  | 1 | Embrechts2023 |  |  | 0.18[0.01,0.36] | 2 | Random | 12.60% |  | 9 |  | Low |
| moderate and vigorous aerobic exercise | the low intensity routine exercises | Stroke | MMSE/MoCA | 1 | Li2024 | 395/391 | SMD | 0.81[0.49,1.13] | 7 | Random | 72%,0.0006 | NA | 8 | Class IV | Moderate |
| ***Non-significant associations*** | | | | | | | | | | | | | | | |
| Combined aerobic and resistance exercise training | nondiet/nonexercise | AD | MMSE | 1 | Panza2018 | NA | SMD | 0.19 [-0.06, 0.43] | 8 | Random | 14.0%;0.320 | <0.01 | 8 | NS | Very low |
| Aerobic exercise/multicomponent exercise/mind-body exercise/resistance exercise | no intervention, usual care, health education, or exercise interventions | Dementia/MCI | Biomarkers of neurotrophy(levels of BDNF) | 1 | Huang2021 | 310(total) | SMD | 0.21[-0.66, 1.08] | 8 | Random | 91.7%,0.000 | NA | 8 | NS | Low |
| regular exercise programs | usual care and without regular exercise | dementia | MMSE | 1 | Li2019 |  | SMD | 0.44[-0.21,1.09] | 15 | Random | 96.7%,<0.001 | 0.355 | 8 | Class IV | Moderate |
| Mind-Body Exercise | nonexercise control | Cognitive Impairment | MMSE | 1 | Wang2018 | 127/121 | SMD | 0.79[-0.09,1.67] | 5 | Random | 90%,<0.00001 | NA | 8 | NS | Low |
| Aerobic exercise | usual care, educational program or other physical training mode except aerobic exercise | Ischemic Cerebrovascular Disorder | MMSE | 1 | Shu2020 | 352/334 | SMD | 0.13[-0.09,0.35] | 7 | Random | 38%.0.14 | NA | 8 | NS | Low |
| Walking | usual physical activities or were administered sham exercises | MCI | MMSE | 1 | Lin2023 | 88/87 | SMD | 0.00[-0.30,0.30] | 3 | Fixed | 0%,1.00 | NA | 10 | NS | Moderate |

AD, Alzheimer's disease; MCI, mild cognitive impairment; PD, Parkinson's Disease; SCD, Subjective Cognitive Decline; VCI, Vscular Cognitive Impairment; MMSE, the Mini Mental State Examination; MoCA, the Montreal Cognitive Assessment; CAMCOG, the Cambridge Cognitive Examination; ADAS-Cog, The Alzheimer’s Disease Assessment Scale–Cognitive Subscale; ERFC, Rapid Assessment of Cognitive Functions test; CMMSE, Cantonese version of Mini Mental Status Examination; CSDD, Cornell Scale for Depression in Dementia; K-MoCA, Korea version of Montreal Cognitive Assessment; MMSE-K, Mini Mental State Examination-Korea version; NCSE, Neurobehavioral Cognitive Status Examination; SMMSE, Standard Mini Mental State Examination; MDRS, Mattis Dementia Rating Scale; SCOPA-COG=Scales for Outcomes in Parkinson's disease-Cognition; PDQ-39, Parkinson’s Disease Questionnaire 39; ACER, Addenbrooke’s Cognitive Examination-Revised; CMT, Cognitive and Motor Therapy; MT, Motor Therapy; CT, Cognitive Therapy; AD, Alzheimer's disease; MCI, mild cognitive impairment; PD, Parkinson's Disease; SCD, Subjective Cognitive Decline; VCI, Vscular Cognitive Impairment
