# Supplementary material for: Effects of exercise interventions on cognitive function in patients with cognitive dysfunction: an umbrella review of meta-analyses
Source: Front Aging Neurosci. 2025 May 16;17:1553868. doi: 10.3389/fnagi.2025.1553868 (PMC12122535; doi:10.3389/fnagi.2025.1553868)
Supplement: Supplementary file 3 [file Data_Sheet_3.doc]

| Supplementary Table S3. Assessments of AMSTAR scores. | | | | | | | | | | | | | | | | |
| --- | --- | --- | --- | --- | --- | --- | --- | --- | --- | --- | --- | --- | --- | --- | --- | --- |
| Exercise intervention | Control | Diseases | Study | A priori design provided | Duplicate study selection & data extraction | At least two electronic databases searched | Status of  publication used as an inclusion criterion | List of  included and excluded studies provided | Characteristics of included  studies provided | Scientific quality of  included studies assessed | Scientific quality of the included studies used  appropriately to form  Conclusions | Appropriate methods to  combine studies | Publication bias assessed | Conflict of interest included | Total AMSTAR Score |  |
| Video Games | a control condition any cognitive stimulation/training technique | AD | Filipa2021 | 1 | 1 | 1 | 0 | 1 | 0 | 1 | 1 | 1 | 1 | 1 | 9 |  |
| aerobic exercise/resistance training | nondiet/nonexercise | AD | Panza2018 | 0 | 1 | 1 | 1 | 1 | 0 | 1 | 0 | 1 | 1 | 1 | 8 |  |
| Exercise | TAU/Daily organized activities/Home safety assessment sessions/Health education classes/Recreational activities/Stretching (HR<50%)/Placebo activity program/Social contacts | AD | Strohle2015 | 1 | 1 | 1 | 0 | 1 | 1 | 1 | 0 | 1 | 1 | 1 | 9 |  |
| Exercise | nonexercise control | AD | Liang2022 | 0 | 1 | 1 | 0 | 1 | 1 | 1 | 0 | 1 | 1 | 1 | 8 |  |
| TCM exercise therapy (Tai Chi, Yi Jin Jing, Ba Duan Jin, Liu Zi Jue, Qigong, Wu Qin Xi etc.) | maintaining the same lifestyle, health education, routine care, low-intensity stretching and stretching exercises. | AD | Guo2024 | 1 | 1 | 1 | 0 | 1 | 1 | 1 | 0 | 1 | 1 | 1 | 9 |  |
| aerobic exercise, athome exercise, Tai Chi | conventional drugs | AD | Roy2023 | 0 | 1 | 1 | 0 | 1 | 1 | 1 | 0 | 1 | 1 | 1 | 8 |  |
| physical activity or exercise carried out at home or delivered via telerehabilitation | usual care or another form of exercise | AD | Abdullahi2024 | 1 | 1 | 1 | 0 | 1 | 1 | 1 | 1 | 1 | 1 | 1 | 10 |  |
| exercise-only intervention | non-diet, non-exercise control group under the guarantee of basically medical care | AD | Jia2019 | 0 | 1 | 1 | 0 | 1 | 1 | 1 | 0 | 1 | 1 | 1 | 8 |  |
| exercise, aerobic activity, physical exercise | social visit or activity, or usual treatment or care | AD | Zeng2023 | 1 | 1 | 1 | 0 | 1 | 1 | 1 | 0 | 1 | 1 | 1 | 9 |  |
| Aerobic exercise |  | AD | Zhou2022 | 0 | 1 | 1 | 0 | 1 | 1 | 1 | 0 | 1 | 1 | 1 | 8 |  |
| Tai Chi and cognitive interventions | Muscle stretching and toning exercise/Attention control/Health education/TAU/Heath advice | Cognitive impairment | Li2022 | 0 | 1 | 1 | 0 | 1 | 1 | 1 | 0 | 1 | 1 | 1 | 8 |  |
| Tai Chi/The combination therapy of Tai Chi and other interventions | N.A./the same other interventions alone | Cognitive impairment | Gu2021 | 1 | 1 | 1 | 0 | 1 | 1 | 1 | 0 | 1 | 1 | 1 | 9 |  |
| movement training based on rhythmic auditory stimulation | conventional treatment, music listening, cognitive training, etc. | cognitive impairment | Wang2024 | 1 | 1 | 1 | 0 | 1 | 1 | 1 | 1 | 1 | 1 | 1 | 10 |  |
| traditional Chinese mind-body exercises | conventional therapy, maintained their daily routine, and did not receive any other exercise therapy | Cognitive impairment | Yao2023 | 1 | 1 | 1 | 0 | 1 | 1 | 1 | 0 | 1 | 1 | 1 | 9 |  |
| Aerobic exercise/multicomponent exercise/mind-body exercise/resistance exercise | no intervention, usual care, health education, or exercise interventions | Dementia | Huang2021 | 1 | 1 | 1 | 0 | 1 | 1 | 0 | 0 | 1 | 1 | 1 | 8 |  |
| aerobic/anaerobic/multicomponent /psychomotor exercise | nonphysical activity or stretching and toning | Cognitive impairment | Sanders2019 | 0 | 1 | 1 | 0 | 1 | 1 | 1 | 0 | 1 | 1 | 1 | 8 |  |
| Multicomponent Exercise | no treatment/usual care/placebo/other conservative treatments | Dementia | Yan2023 | 1 | 1 | 1 | 0 | 1 | 1 | 1 | 0 | 1 | 1 | 1 | 9 |  |
| regular exercise programs | usual care and without regular exercise | dementia | Li2019 | 0 | 1 | 1 | 0 | 1 | 1 | 1 | 0 | 1 | 1 | 1 | 8 |  |
| Mind-Body Exercise | nonexercise control | Dementia | Wang2018 | 0 | 1 | 1 | 0 | 1 | 1 | 1 | 0 | 1 | 1 | 1 | 8 |  |
| exercise, aerobic activity, physical exercise | social visit or activity, or usual treatment or care | Dementia | Zeng2023 | 1 | 1 | 1 | 0 | 1 | 1 | 1 | 0 | 1 | 1 | 1 | 9 |  |
| Exergaming (VR-based, Video-based) | Usual Care, Exercise,Cognitive Training | Dementia | Chan2024 | 1 | 1 | 1 | 0 | 1 | 1 | 1 | 0 | 1 | 1 | 1 | 9 |  |
| Home-Based Physical Activity |  | Dementia | de Almeida2020 | 0 | 1 | 1 | 0 | 1 | 1 | 1 | 0 | 1 | 1 | 1 | 8 |  |
| Exercise/combining physical and cognitive exercises | usual care, social activities, or handicrafts | Dementia | Cardona2021 | 0 | 1 | 1 | 0 | 1 | 1 | 1 | 0 | 1 | 1 | 1 | 8 |  |
| Exercise | nonexercise control | Dementia | Law2020 | 0 | 1 | 1 | 0 | 1 | 1 | 1 | 1 | 1 | 1 | 1 | 9 |  |
| combined cognitive and physical exercise training | Attentioncontrol educational programmes/Sham cognitive and sham exercise/Treatment as usual/Care as usual/Mock-therapy/Psychosocial support/Education control | Dementia | Karssemeijer2017 | 1 | 1 | 1 | 0 | 1 | 1 | 1 | 0 | 1 | 1 | 1 | 9 |  |
| Resistance Training | nonexercise control | Dementia | Coelho-Junior2022 | 0 | 1 | 1 | 0 | 1 | 1 | 1 | 0 | 1 | 1 | 1 | 8 |  |
| Tai Chi | usual care | Dementia | Liu2023 | 0 | 1 | 1 | 0 | 1 | 1 | 1 | 0 | 1 | 1 | 1 | 8 |  |
| aerobic exercise and transcranial direct current stimulation | passive controls | Dementia | Talar2022 | 1 | 1 | 1 | 0 | 1 | 1 | 1 | 0 | 1 | 1 | 1 | 9 |  |
| Aerobic exercise | usual care, educational program or other physical training mode except aerobic exercise | Ischemic Cerebrovascular Disorder | Shu2020 | 0 | 1 | 1 | 0 | 1 | 1 | 1 | 0 | 1 | 1 | 1 | 8 |  |
| Video Games | a control condition any cognitive stimulation/training technique | MCI | Ferreira-Brito2021 | 1 | 1 | 1 | 0 | 1 | 0 | 1 | 1 | 1 | 1 | 1 | 9 |  |
| Aerobic exercise/multicomponent exercise/mind-body exercise/resistance exercise | no intervention, usual care, health education, or exercise interventions | MCI | Huang2021 | 1 | 1 | 1 | 0 | 1 | 1 | 0 | 0 | 1 | 1 | 1 | 8 |  |
| aerobic dance/square dance/ballroom dance/choreographed exercise | Physical therapy/Usual practice/Health education/Blank control/Usual care/Usual lifestyle/Regular care/Medicine | MCI | Yuan2022 | 0 | 1 | 1 | 0 | 1 | 1 | 1 | 0 | 1 | 1 | 1 | 8 |  |
| aerobic exercise/resistance training/mind-body exercise | usual care/lifestyle/sham exercise/health education | MCI | Karamacoska2023 | 1 | 1 | 1 | 0 | 1 | 1 | 1 | 0 | 1 | 1 | 1 | 9 |  |
| Tai Chi and cognitive interventions | Muscle stretching and toning exercise/Attention control/Health education/TAU/Heath advice | MCI | Li2022 | 0 | 1 | 1 | 0 | 1 | 1 | 1 | 0 | 1 | 1 | 1 | 8 |  |
| aerobic/anaerobic/multicomponent /psychomotor exercise | nonphysical activity or stretching and toning | MCI | Sanders2019 | 0 | 1 | 1 | 0 | 1 | 1 | 1 | 0 | 1 | 1 | 1 | 8 |  |
| muscle-strengthening activity/aerobic activity/mind- body activity | health education/social activities/active controls | MCI | Shao2022 | 1 | 1 | 1 | 0 | 1 | 1 | 1 | 0 | 1 | 1 | 1 | 9 |  |
| Multicomponent Exercise | no treatment/usual care/placebo/other conservative treatments | MCI | Yan2023 | 1 | 1 | 1 | 0 | 1 | 1 | 1 | 0 | 1 | 1 | 1 | 9 |  |
| Tai Chi | the conventional exercise group, patients who received education regarding fall prevention and cognition exercise, and the patient group who were given no treatment | MCI | Rampengan2024 | 1 | 1 | 1 | 0 | 1 | 1 | 1 | 0 | 1 | 1 | 1 | 9 |  |
| TCM exercise therapy (Tai Chi, Yi Jin Jing, Ba Duan Jin, Liu Zi Jue, Qigong, Wu Qin Xi etc.) | maintaining the same lifestyle, health education, routine care, low-intensity stretching and stretching exercises. | MCI | Guo2024 | 1 | 1 | 1 | 0 | 1 | 1 | 1 | 0 | 1 | 1 | 1 | 9 |  |
| Aerobic Dance | health education and/or exercise but not aerobic dance training | MCI | Zhu2020 | 0 | 1 | 1 | 0 | 1 | 1 | 1 | 0 | 1 | 1 | 1 | 8 |  |
| Chinese Mind-Body Exercises | active control group (e.g., physical exercise, educational program, social interaction, cognitive training) or passive control group (e.g., usual care, waitlist control, no intervention) were included. | MCI | Ren2021 | 0 | 1 | 1 | 0 | 1 | 1 | 1 | 0 | 1 | 1 | 1 | 8 |  |
| cognitive and physical training | single cognitive or sham intervention (e.g., placebo control, blank control, and passive control)/two or more control groups (e.g., single physical intervention, single cognitive intervention, or sham intervention) | MCI | Han2022 | 1 | 1 | 1 | 0 | 1 | 1 | 1 | 1 | 1 | 1 | 1 | 10 |  |
| Dance | education, walking, waitlisted or no physical activity | MCI | Hewston2021 | 1 | 1 | 1 | 0 | 1 | 1 | 1 | 1 | 1 | 1 | 1 | 10 |  |
| aerobic exercise | exercises of stretching, activities of health education, routine care, daily lifestyle, and social recreation | MCI | Han2023 | 1 | 1 | 1 | 0 | 1 | 0 | 1 | 1 | 1 | 1 | 1 | 9 |  |
| aerobic, resistance, multicomponent, and neuromotor exercises | no treatment, usual care, health education, and stretching | MCI | Ahn2023 | 1 | 1 | 1 | 0 | 1 | 1 | 1 | 0 | 1 | 1 | 1 | 9 |  |
| Mind-Body Exercise | nonexercise control | MCI | Wang2018 | 0 | 1 | 1 | 0 | 1 | 1 | 1 | 0 | 1 | 1 | 1 | 8 |  |
| physical and mental exercises such as taijiquan, Ba Duan Jin, qigong, meditation, yoga, music and dance | conventional care, health education or blank | MCI | Cai2023 | 1 | 1 | 1 | 0 | 1 | 1 | 1 | 0 | 1 | 1 | 1 | 9 |  |
| Traditional Chinese Exercises | usual care, health education ,no intervention, stretching, aerobic exercises | MCI | Zhou2022 | 1 | 1 | 1 | 0 | 1 | 1 | 1 | 0 | 1 | 1 | 1 | 9 |  |
| Physical exercise, breathing exercises, meditation, and yoga-based lifestyle | health education, memory enhancement training, stretching and strengthening exercise, and music listening, no intervention | MCI | Bhattacharyya2021 | 0 | 1 | 1 | 0 | 1 | 1 | 1 | 0 | 1 | 1 | 1 | 8 |  |
| Exergaming (VR-based, Video-based) | Usual Care, Exercise,Cognitive Training | MCI | Chan2024 | 1 | 1 | 1 | 0 | 1 | 1 | 1 | 0 | 1 | 1 | 1 | 9 |  |
| dance/simultaneous multicomponent exercise/momentum-dumbbell training program/exercise training technology | passive control conditions/health and/or education classes | MCI | Zawaly2022 | 0 | 1 | 1 | 0 | 1 | 1 | 1 | 0 | 1 | 1 | 1 | 8 |  |
| Resistance Training | routine lifestyle without any exercise activities, balance and tone exercise, and sham training similar to the resistance training | MCI | Zhang2020 | 0 | 1 | 1 | 0 | 1 | 1 | 1 | 0 | 1 | 1 | 1 | 8 |  |
| walking | usual physical activities or were administered sham exercises | MCI | Lin2023 | 1 | 1 | 1 | 0 | 1 | 1 | 1 | 1 | 1 | 1 | 1 | 10 |  |
| Exercise | no treatment, waitlist control, relaxation | MCI | Liu2023 | 1 | 1 | 1 | 0 | 1 | 1 | 1 | 1 | 1 | 1 | 1 | 10 |  |
| Exercise | health education or maintains their current way of life | MCI | Liu2024 | 1 | 1 | 1 | 0 | 1 | 1 | 1 | 0 | 1 | 1 | 1 | 9 |  |
| Baduanjin | conventional therapy, maintained their daily routine, and did not receive other exercise therapy | MCI | Yu2021 | 0 | 1 | 1 | 0 | 1 | 1 | 1 | 0 | 1 | 1 | 1 | 8 |  |
| aerobic exercise and transcranial direct current stimulation | passive controls | MCI | Talar2022 | 1 | 1 | 1 | 0 | 1 | 1 | 1 | 0 | 1 | 1 | 1 | 9 |  |
| aerobic, resistance and multimodal exercises |  | MCI | Akalp2024 | 0 | 1 | 1 | 0 | 1 | 1 | 1 | 0 | 1 | 1 | 1 | 8 |  |
| Exercise | nonexercise control | PD | Folkerts2024 | 1 | 1 | 1 | 0 | 1 | 1 | 1 | 1 | 1 | 1 | 1 | 10 |  |
| aerobic/strength/balance exercise/flexibility exercise/combined exercise | Usual care/No intervention/Stretching/Wait-list | PD | Kim2023 | 1 | 1 | 1 | 0 | 1 | 1 | 1 | 0 | 1 | 1 | 1 | 9 |  |
| Mind-Body Exercises | usual care, no intervention, placebo, or routine physiotherapy exercises | PD | Wang2021 | 1 | 1 | 1 | 0 | 1 | 1 | 1 | 0 | 1 | 1 | 1 | 9 |  |
| Tai Chi | other training forms, usual healthcare, or no intervention | PD | Yin2023 | 0 | 1 | 1 | 0 | 1 | 1 | 1 | 0 | 1 | 1 | 1 | 8 |  |
| Aerobic exercise |  | Post-stroke | Li2022 | 0 | 1 | 1 | 1 | 1 | 1 | 1 | 0 | 1 | 1 | 1 | 9 |  |
| resistance training, flexibility training, aerobic training, and mixed training combined with multiple exercises | outine care, conventional physiotherapy, health education, or no treatment | Post-stroke | Zhao2024 | 1 | 1 | 1 | 0 | 1 | 1 | 1 | 0 | 1 | 1 | 1 | 9 |  |
| aerobic exercise, resistance exercise, and multiple combination exercises | routine non-pharmacological intervention, including a balanced diet, health education, and routine rehabilitation training | Post-stroke | Zhang2023 | 1 | 1 | 1 | 0 | 1 | 1 | 1 | 0 | 1 | 1 | 1 | 9 |  |
| Exercise | nonexercise control | Post-stroke | Hernandez2021 | 0 | 1 | 1 | 0 | 1 | 1 | 1 | 0 | 1 | 1 | 1 | 8 |  |
| CMT | MT/CT/No Therapy | stroke | Embrechts2023 | 1 | 1 | 1 | 0 | 1 | 1 | 1 | 0 | 1 | 1 | 1 | 9 |  |
| moderate and vigorous aerobic exercise | the low intensity routine exercises | stroke | Li2024 | 1 | 1 | 1 | 0 | 1 | 0 | 1 | 0 | 1 | 1 | 1 | 8 |  |
| aerobic exercise/resistance training/mind-body exercise | usual care/lifestyle/sham exercise/health education | VCI | Karamacoska2023 | 1 | 1 | 1 | 0 | 1 | 1 | 1 | 0 | 1 | 1 | 1 | 9 |  |
| aerobic/anaerobic/multicomponent /psychomotor exercise | nonphysical activity or stretching and toning | VCI | Sanders2019 | 0 | 1 | 1 | 0 | 1 | 1 | 1 | 0 | 1 | 1 | 1 | 8 |  |
| aerobic exercise/resistance training/mind-body exercise | usual care/lifestyle/sham exercise/health education | SCD | Karamacoska2023 | 1 | 1 | 1 | 0 | 1 | 1 | 1 | 0 | 1 | 1 | 1 | 9 |  |
| AD, Alzheimer's disease; MCI, mild cognitive impairment; PD, Parkinson's Disease; VCI, vascular cognitive impairment; TCM, traditional chinese medicine; CMT, Cognitive and Motor Therapy; MT, Motor Therapy; CT, Cognitive Therapy | | | | | | | | | | | | | | | | |
