# Supplementary material for: Effects of exercise interventions on cognitive function in patients with cognitive dysfunction: an umbrella review of meta-analyses
Source: Front Aging Neurosci. 2025 May 16;17:1553868. doi: 10.3389/fnagi.2025.1553868 (PMC12122535; doi:10.3389/fnagi.2025.1553868)
Supplement: Supplementary file 4 [file Data_Sheet_4.doc]

Table S4. GRADE classification of quality of evidence.

| Exercise intervention | Control | Diseases | Outcomes | Study | No. of studies | Risk of bias | Inconsistency I²>50%,P<0.1 | Indirectness | Imprecision P<0.001 | Publication bias egger<0.05 | Plausible confounding | Magnitude of effect | Dose-response gradient | Quality |
| --- | --- | --- | --- | --- | --- | --- | --- | --- | --- | --- | --- | --- | --- | --- |
| Video Games | a control condition any cognitive stimulation/training technique | AD/MCI | MMSE | Ferreira-Brito2021 | 4 | serious risk | no serious inconsistency | no serious  indirectness | serious imprecision | NA | would not reduce effect | no | no | Low |
| Aerobic exercise |  | AD | MMSE | Zhou2022 | 12 | no serious risk | serious inconsistency | no serious  indirectness | no serious imprecision | undetected | would not reduce effect | no | no | Moderate |
| Exercise | TAU/Daily organized activities/Home safety assessment sessions/Health education classes/Recreational activities/Stretching (HR<50%)/Placebo activity program/Social contacts | AD | the cognitive subscale of the Alzheimer Disease Assessment Scale, the Severe Impairment Battery (for Mini-Mental State Exam [MMSE] score <10), MMSE, or SKT | Strohle2015 | 4 | no serious risk | serious inconsistency | no serious  indirectness | no serious imprecision | undetected | would not reduce effect | no | no | Moderate |
| Exercise | TAU/Daily organized activities/Home safety assessment sessions/Health education classes/Recreational activities/Stretching (HR<50%)/Placebo activity program/Social contacts | MCI | the cognitive subscale of the Alzheimer Disease Assessment Scale, the Severe Impairment Battery (for Mini-Mental State Exam [MMSE] score <10), MMSE, or SKT | Strohle2015 | 6 | no serious risk | serious inconsistency | no serious  indirectness | no serious imprecision | undetected | would not reduce effect | no | no | Moderate |
| Exercise | nonexercise control | AD | MMSE | Liang2022 | NA | no serious risk | serious inconsistency | no serious  indirectness | no serious imprecision | NA | would not reduce effect | no | no | Moderate |
| TCM exercise therapy | maintaining the same lifestyle, health education, routine care, low-intensity stretching and stretching exercises. | AD/MCI | MMSE/MoCA | Guo2024 | 20 | serious risk | serious inconsistency | no serious  indirectness | no serious imprecision | strongly suspected | would not reduce effect | no | no | Very low |
| aerobic exercise, athome exercise, Tai Chi | conventional drugs | AD | MMSE | Roy2023 | 7 | no serious risk | serious inconsistency | no serious  indirectness | serious imprecision | undetected | would not reduce effect | no | no | Low |
| physical activity or exercise carried out at home or delivered via telerehabilitation | usual care or another form of exercise | AD | MMSE | Abdullahi2024 | 3 | serious risk | no serious inconsistency | no serious  indirectness | serious imprecision | NA | would not reduce effect | no | no | Low |
| exercise-only intervention | non-diet, non-exercise control group under the guarantee of basically medical care | AD | MMSE | Jia2019 | 13 | no serious risk | serious inconsistency | no serious  indirectness | no serious imprecision | undetected | would not reduce effect | no | no | Moderate |
| exercise, aerobic activity, physical exercise | social visit or activity, or usual treatment or care | AD/Dementia |  | Zeng2023 | 18 | serious risk | serious inconsistency | no serious  indirectness | serious imprecision | strongly suspected | would not reduce effect | no | no | Very low |
| Tai Chi and cognitive interventions | Muscle stretching and toning exercise/Attention control/Health education/TAU/Heath advice | Cognitive impairment/MCI | MMSE/MoCA/ADAS-Cog/Clinical Dementia Rating/Dementia Rating Scale | Li2022 | 6 | no serious risk | serious inconsistency | no serious  indirectness | serious imprecision | NA | would not reduce effect | no | no | Low |
| Tai Chi/The combination therapy of Tai Chi and other interventions | NA/the same other interventions alone | Cognitive impairment | MMSE | Gu2021 | 6 | no serious risk | serious inconsistency | no serious  indirectness | no serious imprecision | NA | would not reduce effect | no | no | Moderate |
| movement training based on rhythmic auditory stimulation | conventional treatment, music listening, cognitive training, etc. | Cognitive impairment | MMSE | Wang2024 | 6 | no serious risk | serious inconsistency | no serious  indirectness | serious imprecision | NA | would not reduce effect | no | no | Low |
| traditional Chinese mind-body exercises | conventional therapy, maintained their daily routine, and did not receive any other exercise therapy | Cognitive impairment | MoCA | Yao2023 | 9 | no serious risk | no serious inconsistency | no serious  indirectness | no serious imprecision | undetected | would not reduce effect | no | no | High |
| aerobic/anaerobic/multicomponent /psychomotor exercise | nonphysical activity or stretching and toning | Dementia/MCI/VCI | MMSE | Sanders2019 | 9 | no serious risk | serious inconsistency | no serious  indirectness | serious imprecision | undetected | would not reduce effect | no | no | Low |
| Multicomponent Exercise | no treatment/usual care/placebo/other conservative treatments | Dementia | MMSE/ERFC/ADAS-cog | c2023 | 9 | no serious risk | serious inconsistency | no serious  indirectness | serious imprecision | strongly suspected | would not reduce effect | no | no | Very low |
| Mind-Body Exercise | nonexercise control | MCI | MMSE | Wang2018 | 4 | no serious risk | serious inconsistency | no serious  indirectness | serious imprecision | undetected | would not reduce effect | no | no | Low |
| Exergaming (VR-based, Video-based) | Usual Care, Exercise,Cognitive Training | MCI | MMSE/MoCA | Chan2024 | 10 | no serious risk | no serious inconsistency | no serious  indirectness | no serious imprecision | undetected | would not reduce effect | no | no | High |
| Exergaming (VR-based, Video-based) | Usual Care, Exercise,Cognitive Training | Dementia | MMSE/MoCA | Chan2024 | 4 | no serious risk | serious inconsistency | no serious  indirectness | serious imprecision | undetected | would not reduce effect | no | no | Low |
| Home-Based Physical Activity |  | Dementia | MMSE | de Almeida2020 | 3 | no serious risk | serious inconsistency | no serious  indirectness | no serious imprecision | NA | would not reduce effect | no | no | Moderate |
| Exercise/combining physical and cognitive exercises | usual care, social activities, or handicrafts | Dementia | MMSE/ADAS-Cog | Cardona2021 | 16 | no serious risk | serious inconsistency | no serious  indirectness | serious imprecision | strongly suspected | would not reduce effect | no | no | Very low |
| Exercise | nonexercise control | Dementia/MCI | MMSE | Law2020 | 26 | no serious risk | serious inconsistency | no serious  indirectness | serious imprecision | undetected | would not reduce effect | no | no | Low |
| combined cognitive and physical exercise training | Attentioncontrol educational programmes/Sham cognitive and sham exercise/Treatment as usual/Care as usual/Mock-therapy/Psychosocial support/Education control | Dementia/MCI | MMSE/ADAS-Cog | Karssemeije2017 | 10 | no serious risk | no serious inconsistency | no serious  indirectness | no serious imprecision | undetected | would not reduce effect | no | no | High |
| Resistance Training | nonexercise control | Dementia | MMSE/MoCA/CAMCOG | Coelho-Junior2022 | 4 | no serious risk | serious inconsistency | no serious  indirectness | no serious imprecision | NA | would not reduce effect | no | no | Moderate |
| Tai Chi | usual care | Dementia | MMSE | Liu2023 | 3 | no serious risk | no serious inconsistency | no serious  indirectness | serious imprecision | undetected | would not reduce effect | no | no | Moderate |
| aerobic exercise and transcranial direct current stimulation | aerobic exercise and transcranial direct current stimulation | Healthy, MCI and Dementia | MMSE/MoCA/CAMCOG | Talar2022 | 20 | no serious risk | serious inconsistency | no serious  indirectness | no serious imprecision | NA | would not reduce effect | no | no | Moderate |
| aerobic dance/square dance/ballroom dance/choreographed exercise | Physical therapy/Usual practice/Health education/Blank control/Usual care/Usual lifestyle/Regular care/Medicine | MCI | MMSE | Yuan2022 | 8 | no serious risk | serious inconsistency | no serious  indirectness | serious imprecision | undetected | would not reduce effect | no | no | Low |
| aerobic exercise/resistance training/mind-body exercise | usual care/lifestyle/sham exercise/health education | MCI/SCD/VCI | MMSE/MoCA/ADAS-Cog | Karamacoska2023 | 13 | no serious risk | serious inconsistency | no serious  indirectness | no serious imprecision | undetected | would not reduce effect | no | no | Moderate |
| muscle-strengthening activity/aerobic activity/mind- body activity | health education/social activities/active controls | MCI | MMSE | Shao2022 | 24 | serious risk | serious inconsistency | no serious  indirectness | no serious imprecision | undetected | would not reduce effect | no | no | Low |
| Multicomponent Exercise | no treatment/usual care/placebo/other conservative treatments | MCI | MMSE/MoCA/ADAS-Cog | Yan2023 | 10 | no serious risk | serious inconsistency | no serious  indirectness | serious imprecision | undetected | would not reduce effect | no | no | Low |
| Tai Chi | the conventional exercise group, patients who received education regarding fall prevention and cognition exercise, and the patient group who were given no treatment | MCI | MMSE | Rampengan2024 | 5 | no serious risk | no serious inconsistency | no serious  indirectness | no serious imprecision | NA | would not reduce effect | no | no | High |
| Aerobic Dance | health education and/or exercise but not aerobic dance training | MCI | MMSE | Zhu2020 | 3 | no serious risk | serious inconsistency | no serious  indirectness | no serious imprecision | NA | would not reduce effect | no | no | Moderate |
| Chinese Mind-Body Exercises | active control group (e.g., physical exercise, educational program, social interaction, cognitive training) or passive control group (e.g., usual care, waitlist control, no intervention) were included. | MCI | (EF) | Ren2021 | 29 | no serious risk | serious inconsistency | no serious  indirectness | no serious imprecision | undetected | would not reduce effect | no | no | Moderate |
| cognitive and physical training | single cognitive or sham intervention (e.g., placebo control, blank control, and passive control)/two or more control groups (e.g., single physical intervention, single cognitive intervention, or sham intervention) | MCI | MMSE | Han2022 | 3 | no serious risk | serious inconsistency | no serious  indirectness | no serious imprecision | NA | would not reduce effect | no | no | Moderate |
| Dance | education, walking, waitlisted or no physical activity | MCI | MMSE | Hewston2021 | 2 | no serious risk | serious inconsistency | no serious  indirectness | serious imprecision | NA | would not reduce effect | no | no | Low |
| Aerobic exercise | exercises of stretching, activities of health education, routine care, daily lifestyle, and social recreation | MCI | MMSE | Han2023 | 14 | no serious risk | serious inconsistency | no serious  indirectness | no serious imprecision | NA | would not reduce effect | no | no | Moderate |
| aerobic, resistance, multicomponent, and neuromotor exercises | no treatment, usual care, health education, and stretching | MCI | MMSE/MoCA/CMMSE/K-MoCA/MMSE-K/NCSE/SMMSE | Ahn2023 | 21 | no serious risk | serious inconsistency | no serious  indirectness | no serious imprecision | undetected | would not reduce effect | no | no | Moderate |
| physical and mental exercises such as taijiquan, Ba Duan Jin, qigong, meditation, yoga, music and dance | conventional care, health education or blank | MCI | MMSE | Cai2023 | 14 | no serious risk | serious inconsistency | no serious  indirectness | serious imprecision | undetected | would not reduce effect | no | no | Low |
| Traditional Chinese Exercises | usual care, health education ,no intervention, stretching, aerobic exercises | MCI | MMSE/MoCA/Cogntive adatptations | Zhou2022 | 7 | no serious risk | no serious inconsistency | no serious  indirectness | no serious imprecision | undetected | would not reduce effect | no | no | High |
| dance/simultaneous multicomponent exercise/momentum-dumbbell training program/exercise training technology | passive control conditions/health and/or education classes | MCI | MMSE | Zawaly2022 | 4 | no serious risk | no serious inconsistency | no serious  indirectness | serious imprecision | NA | would not reduce effect | no | no | Moderate |
| Resistance Training | routine lifestyle without any exercise activities, balance and tone exercise, and sham training similar to the resistance training | MCI | MMSE/MoCA/ADAS-Cog | Zhang2020 | 5 | no serious risk | serious inconsistency | no serious  indirectness | serious imprecision | NA | would not reduce effect | no | no | Low |
| Exercise | no treatment, waitlist control, relaxation | MCI | MMSE/MoCA/ADAS-Cog | Liu2023 | 25 | no serious risk | serious inconsistency | no serious  indirectness | no serious imprecision | undetected | would not reduce effect | no | no | Moderate |
| Exercise | health education or maintains their current way of life | MCI | MMSE/MoCA | Liu2024 | 20 | no serious risk | serious inconsistency | no serious  indirectness | no serious imprecision | undetected | would not reduce effect | no | no | Moderate |
| Baduanjin | conventional therapy, maintained their daily routine, and did not receive other exercise therapy | MCI | MoCA-BJ | Yu2021 | 10 | no serious risk | serious inconsistency | no serious  indirectness | no serious imprecision | NA | would not reduce effect | no | no | Moderate |
| aerobic, resistance and multimodal exercises |  | MCI | MMSE/MoCA | Akalp2024 | 18 | no serious risk | serious inconsistency | no serious  indirectness | no serious imprecision | undetected | would not reduce effect | no | no | Moderate |
| Exercise | passive and active control groups | PD | MMSE/MoCA/MDRS | Folkerts2024 | 9 | no serious risk | no serious inconsistency | no serious  indirectness | serious imprecision | undetected | would not reduce effect | no | no | Moderate |
| aerobic/strength/balance exercise/flexibility exercise/combined exercise | Usual care/No intervention/Stretching/Wait-list | PD | MMSE/MoCA/SCOPA-COG | Kim2023 | 16 | no serious risk | serious inconsistency | no serious  indirectness | no serious imprecision | strongly suspected | would not reduce effect | no | no | Low |
| Mind-Body Exercises | usual care, no intervention, placebo, or routine physiotherapy exercises | PD | MoCA | Wang2021 | 9 | no serious risk | serious inconsistency | no serious  indirectness | no serious imprecision | undetected | would not reduce effect | no | no | Moderate |
| Tai Chi | other training forms, usual healthcare, or no intervention | PD | MoCA/K-MMSE/PDQ-39 | Yin2023 | 6 | no serious risk | serious inconsistency | no serious  indirectness | serious imprecision | NA | would not reduce effect | no | no | Low |
| Aerobic exercise |  | Post-stroke | MMSE/MoCA | Li2022 | 6 | no serious risk | serious inconsistency | no serious  indirectness | serious imprecision | NA | would not reduce effect | no | no | Low |
| resistance training, flexibility training, aerobic training, and mixed training combined with multiple exercises | outine care, conventional physiotherapy, health education, or no treatment | Post-stroke | MMSE/MoCA/ACER | Zhao2024 | 18 | no serious risk | serious inconsistency | no serious  indirectness | no serious imprecision | undetected | would not reduce effect | no | no | Moderate |
| aerobic exercise, resistance exercise, and multiple combination exercises | routine non-pharmacological intervention, including a balanced diet, health education, and routine rehabilitation training | Post-stroke | MMSE/MoCA | Zhang2023 | 10 | no serious risk | serious inconsistency | no serious  indirectness | no serious imprecision | undetected | would not reduce effect | no | no | Moderate |
| Exercise | nonexercise control | Stroke | MMSE/MoCA | Hernandez2021 | 9 | no serious risk | serious inconsistency | no serious  indirectness | no serious imprecision | strongly suspected | would not reduce effect | no | no | Low |
| CMT | No Therapy | Stroke |  | Embrechts2023 | 7 | no serious risk | serious inconsistency | no serious  indirectness |  | NA | would not reduce effect | no | no |  |
|  | MT |  |  |  | 18 | no serious risk | serious inconsistency | no serious  indirectness |  | NA | would not reduce effect | no | no |  |
|  | CT |  |  |  | 2 | no serious risk | no serious inconsistency | no serious  indirectness |  | NA | would not reduce effect | no | no |  |
| moderate and vigorous aerobic exercise | the low intensity routine exercises | Stroke | MMSE/MoCA | Li2024 | 7 | no serious risk | serious inconsistency | no serious  indirectness | no serious imprecision | NA | would not reduce effect | no | no | Moderate |
| Combined aerobic and resistance exercise training | nondiet/nonexercise | AD | MMSE | Panza2018 | 8 | no serious risk | serious inconsistency | no serious  indirectness | serious imprecision | strongly suspected | would not reduce effect | no | no | Very low |
| Aerobic exercise/multicomponent exercise/mind-body exercise/resistance exercise | no intervention, usual care, health education, or exercise interventions | Dementia/MCI | Biomarkers of neurotrophy(levels of BDNF) | Huang2021 | 8 | no serious risk | serious inconsistency | no serious  indirectness | serious imprecision | NA | would not reduce effect | no | no | Low |
| regular exercise programs | usual care and without regular exercise | dementia | MMSE | Li2019 | 15 | no serious risk | serious inconsistency | no serious  indirectness | no serious imprecision | undetected | would not reduce effect | no | no | Moderate |
| Mind-Body Exercise | nonexercise control | Cognitive Impairment | MMSE | Wang2018 | 5 | no serious risk | serious inconsistency | no serious  indirectness | serious imprecision | undetected | would not reduce effect | no | no | Low |
| Aerobic exercise | usual care, educational program or other physical training mode except aerobic exercise | Ischemic Cerebrovascular Disorder | MMSE | Shu2020 | 7 | no serious risk | serious inconsistency | no serious  indirectness | serious imprecision | NA | would not reduce effect | no | no | Low |
| Walking | usual physical activities or were administered sham exercises | MCI | MMSE | Lin2023 | 3 | no serious risk | no serious inconsistency | no serious  indirectness | serious imprecision | NA | would not reduce effect | no | no | Moderate |

AD, Alzheimer's disease; MCI, mild cognitive impairment; PD, Parkinson's Disease; SCD, Subjective Cognitive Decline; VCI, Vscular Cognitive Impairment; MMSE, the Mini Mental State Examination; MoCA, the Montreal Cognitive Assessment; CAMCOG, the Cambridge Cognitive Examination; ADAS-Cog, The Alzheimer’s Disease Assessment Scale–Cognitive Subscale; ERFC, Rapid Assessment of Cognitive Functions test; CMMSE, Cantonese version of Mini Mental Status Examination; CSDD, Cornell Scale for Depression in Dementia; K-MoCA, Korea version of Montreal Cognitive Assessment; MMSE-K, Mini Mental State Examination-Korea version; NCSE, Neurobehavioral Cognitive Status Examination; SMMSE, Standard Mini Mental State Examination; MDRS, Mattis Dementia Rating Scale; SCOPA-COG=Scales for Outcomes in Parkinson's disease-Cognition; PDQ-39, Parkinson’s Disease Questionnaire 39; ACER, Addenbrooke’s Cognitive Examination-Revised; CMT, Cognitive and Motor Therapy; MT, Motor Therapy; CT, Cognitive Therapy; AD, Alzheimer's disease; MCI, mild cognitive impairment; PD, Parkinson's Disease; SCD, Subjective Cognitive Decline; VCI, Vscular Cognitive Impairment
